# Cleavage of histone H2A during embryonic stem cell differentiation destabilizes nucleosomes to counteract gene activation

**DOI:** 10.1016/j.jbc.2026.111437

**Published:** 2026-04-09

**Authors:** Mariel Coradin, Elizabeth G. Porter, Francisca Nathalia Vitorino, Richard M. Searfoss, Joseph Cesare, Yemin Lan, Zhexin Zhu, Peder J. Lund, Simone Sidoli, Yekaterina Perez, Congcong Lu, Justin Brumbaugh, Charles W.M. Roberts, Benjamin A. Garcia

**Affiliations:** 1 Biochemistry and Molecular Biophysics Graduate Group, University of Pennsylvania, Philadelphia, Pennsylvania, USA; 2 Department of Biochemistry and Biophysics, University of Pennsylvania, Philadelphia, Pennsylvania, USA; 3 Epigenetics Institute, Perelman School of Medicine, University of Pennsylvania, Philadelphia, Pennsylvania, USA; 4 Department of Biochemistry, Albert Einstein College of Medicine, Bronx, New York, USA; 5 Department of Oncology, St Jude Children's Research Hospital, Memphis, Tennessee, USA; 6 Department of Biochemistry and Molecular Biophysics Washington University in St Louis, St Louis, Missouri, USA; 7 Department of Molecular, Cellular, and Developmental Biology, University of Colorado Boulder, Boulder Colorado, USA

**Keywords:** proteolysis, histone, stem cell, mass spectrometry, proteomics, ChIP-seq, differentiation, epigenetic, chromatin remodeler

## Abstract

Histone proteolysis is an understudied phenomenon in which the N-terminal tails of histones are irreversibly cleaved by intracellular proteases. During development, histone post-translational modifications (PTMs) are known to orchestrate gene expression patterns that ultimately drive cell fate decisions. Therefore, deciphering the mechanisms of histone proteolysis is necessary to enhance the understanding of cellular differentiation. Here, we show that histone H2A is cleaved by the lysosomal protease Cathepsin L during mouse ESC differentiation. Using quantitative mass spectrometry (MS), we identified L23 to be the primary cleavage site that gives rise to the main clipped form of H2A (cH2A), which reaches a maximum level of ∼1% of total H2A after 4 days of ESC differentiation. Using ChIP-seq, we found that preventing proteolysis leads to an increase in acetylated H2A at promoter regions in differentiated ES cells. We also identified novel readers of different acetylated forms of H2A in pluripotent ES cells, such as members of the PBAF remodeling complex, and showed that H2A proteolysis abolishes this recognition. Analysis of the histone H3 PTM profiles of full-length (FL) H2A and cH2A containing nucleosomes demonstrates that cH2A is associated with marks found on active genes, consistent with ChIP-seq experiments. cH2A-containing nucleosomes are also less stable or turned over at faster rates than nucleosomes containing FL H2A. Altogether, our data suggest that proteolysis serves as an efficient mechanism to silence pluripotency genes and destabilize the nucleosome core particle.

Eukaryotic cells condense their DNA around histone proteins to form chromatin. The basic repeating unit of chromatin is the nucleosome, consisting of approximately 150 base pairs (bp) of DNA wrapped around a histone octamer composed of two copies of each histone H3, H4, H2A, and H2B (1). Extending out from the nucleosome core, histone tails (residues ∼1–50) are the major acceptor of post-translational modifications (PTMs), among which acetylation, methylation, and phosphorylation are the most common. Different biological outcomes are associated with the specific sites and types of histone PTMs, such as gene activation for H3K27ac, gene silencing for H3K9me3, and mitosis for H3S10ph (2). The proper regulation of gene expression by histone PTMs is crucial for guiding cellular differentiation and dictating cell fate.

Embryonic stem (ES) cell differentiation gives rise to the three germ layers, the endoderm, mesoderm, and ectoderm, which are critical for development (3). This process of differentiation is tightly regulated by external cues as well as internal machinery, such as transcription factors and epigenetic modulators, which have been shown to remodel chromatin and alter gene expression during cell fate commitment (4). During lineage commitment, cells experience major changes in chromatin architecture, resulting in alteration of gene expression (5, 6). As cells differentiate, chromatin becomes less accessible and more compact with decreased physical elasticity (7). These changes are driven in part by chromatin remodelers acting to reposition nucleosomes and/or promote histone variant exchange (8). Differentiation also leads to changes in histone PTM patterns (6). For instance, during the early stages of differentiation, ES cells show high levels of acetylation, a mark of active chromatin, while the heterochromatic mark H3K9me3 is at low levels in pluripotent ES cells (9).

Histone proteolysis, another potential form of epigenetic regulation, is a lesser explored process in which histone tails are enzymatically cleaved, thereby irreversibly removing existing PTMs, precluding future modifications until histone exchange occurs. Histone proteolysis has been observed in different biological contexts. H2A, for example, has been shown to undergo proteolysis in acute monocytic leukemia at residue V114 (10). Similarly, H2B has been found to be cleaved in hepatocytes (11). Histone clipping appears to be dictated not only by the primary amino acid sequence but also by the PTMs on histone tails. Jumonji-C domain-containing proteins JMJD5 and JMJD7 have been shown to cleave methylated arginine in mouse embryonic fibroblasts (MEFs) (12). Furthermore, during osteoclastogenesis, H3K18ac has been shown to modulate proteolysis of histone H3 by matrix metalloproteinase 9 (MMP-9) (13).

In the context of cellular development, the lysosomal protease Cathepsin L (CTSL) has been shown to cleave H3 during ESC differentiation (14). CTSL localizes to the nucleus upon Ras activation, where it is known to cleave the CDP/Cux transcription factor, regulating the cell cycle (15). Additionally, CTSL has been shown to promote cellular senescence in IMR90 fibroblasts and primary melanocytes by cleaving the histone H3.3 variant (16). CTSL knockout embryos exhibit abnormal visceral endoderm formation (17). Furthermore, CTSL-deficient mice show irregular hair follicle development and other skin pathologies (18). Prior to our work, the biological significance of histone proteolysis by CTSL in regulating gene expression during stem cell differentiation had remained unresolved. Additionally, questions on whether this protease cleaves other histones and whether this processing affects nucleosome stability was yet to be investigated.

Here, we report that CTSL cleaves histone H2A during embryonic stem cell differentiation. We hypothesized that H2A proteolysis serves as a rapid mechanism to remove N-terminal histone H2A modifications, thus counteracting gene activation during development. Using high-resolution quantitative mass spectrometry (qMS), we localized the primary cleavage sites to lie between amino acids 22 and 25. Additionally, we found that CTSL knockdown leads to a global increase in H2A N-terminal acetylation after 4 days of differentiation. Using chromatin immunoprecipitation followed by next-generation sequencing (ChIP-seq), we determined that acetylated H2A is redistributed to promoter regions upon CTSL knockdown. We have also discovered that members of the SWI/SNF remodeling complex bind to acetylated H2A but not to cleaved H2A (cH2A). Lastly, we observed that nucleosomes containing cH2A harbor different histone H3 PTM patterns and are less stable than full-length H2A (FL-H2A). Taken together, our results uncover cellular consequences of histone proteolysis and describe the role of this cleavage in altering H2A modifications, gene expression patterns and nucleosome stability during cell fate commitment.

## Results

### The N-terminal tail of histone H2A is cleaved during mouse embryonic stem cell differentiation

**Figure 1:**
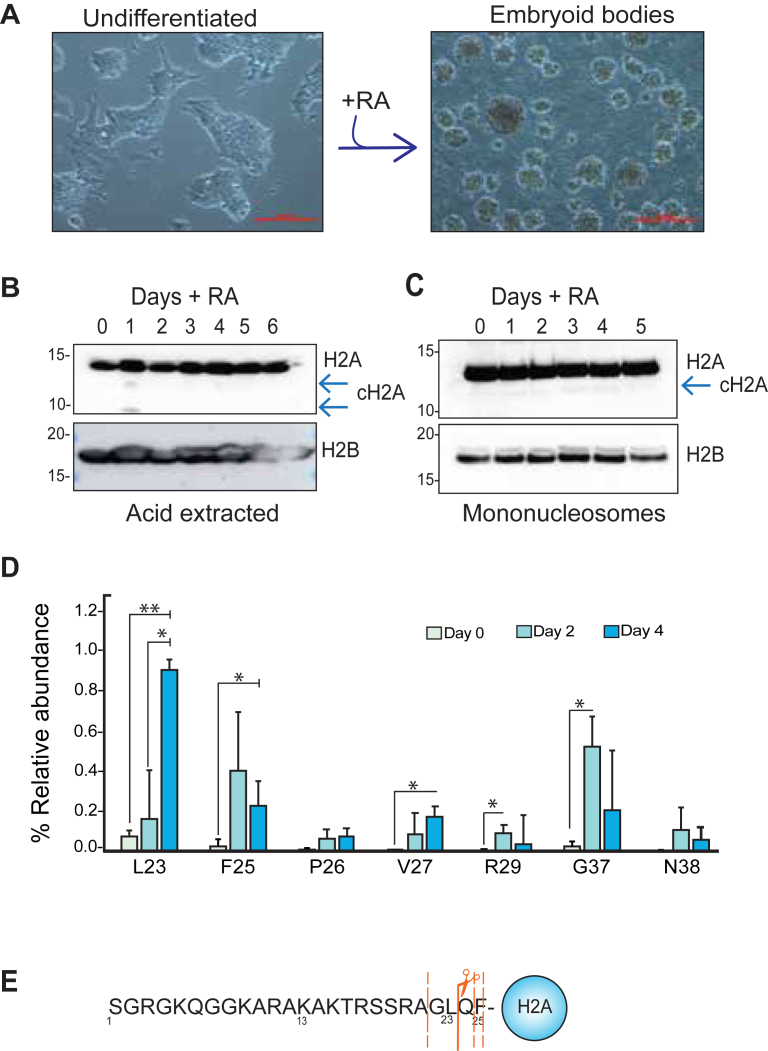
**Histone H2A is cleaved during ES cell differentiation.***A*, Undifferentiated ES cells, (*left* panel) differentiated into embryoid bodies (*right* panel) after treatment with retinoic acid (RA) treatment (1000 μm scale bar). Western blot analysis of (*B*) acid extracted histones or (*C*) mononucleosomes during 6 or 5 days of differentiation, *arrow* indicates the presence of cleaved H2A (cH2A). *D*, mass Spectrometry quantification of cH2A relative abundance compared to FL-H2A. *Asterisks* indicate significant differences between undifferentiated (Day 0) and differentiated cells (Day 2 and 4), two-tailed Student's *t* test (∗*p*< 0.05, ∗∗*p*< 0.01). *Bar* plots represent the average of three biological replicates, and the error bars represent the S.D. *E*, illustration of the N-terminal tail of H2A, *dotted lines* indicate secondary cleavage sites while the *solid line* denotes the primary and most abundant cleavage site (L23).

Prior studies have reported that histone H3 can be cleaved upon cellular differentiation (14). To determine if differentiation involves the cleavage of other histones, we treated mouse embryonic stem cells (mESCs) with retinoic acid (RA) to induce their differentiation into embryoid bodies (EBs, Fig. 1*A*). Using immunoblot analysis, we identified that histone H2A is cleaved at a very low abundance upon differentiation at Day 1 and 4 (Fig. 1*B*), and no change was observed for H2B. Next, we sought to interrogate whether cleaved H2A (cH2A) is still associated with chromatin by isolating mononucleosomes from both undifferentiated mESCs and EBs using micrococcal nuclease (MNase) digestion. As shown in Figure 1*C*, cH2A is present in mononucleosomes derived from EBs, but at much lower levels in undifferentiated cells (consistent with our results in Fig. 1*B*), suggesting that cH2A is chromatin-associated.

Next, we utilized quantitative top-down mass spectrometry to identify the cleavage site(s) on histone H2A and much more precisely quantify the levels of cH2A upon differentiation. Histones from differentiated and undifferentiated mESCs were extracted with sulfuric acid and then further purified by reverse-phase liquid chromatography (RP-HPLC). Characteristic fractions corresponding to canonical H2A (19) (Fig. S1*A*) were analyzed by liquid chromatography coupled to MS (LC-MS) on an Orbitrap mass spectrometer. In agreement with our immunoblot data, we detected increasing levels of cleavage of H2A at Day 2 of differentiation, which increased significantly further by Day 4 of RA treatment. The most abundant cleavage site we identified was at L23, with ∼1% of all H2A being cleaved at this site (Fig. 1, *D* and *E*; and Data S1). Other cleavage sites that increased through differentiation were also identified at F25, V27 and G37. Although additional cleavage sites were detected along the H2A tail (Fig. S1, *B*–*D*), their abundance did not change during differentiation, indicating that these sites were not regulated during cell fate transitions. Numerous variants of H2A exist, including macroH2A, H2A.Z, and H2A.X, which have different roles in cellular differentiation (20). Although addressing whether other specialized H2A variants undergo cleavage during development is beyond the scope of this work, sequence alignment shows that the cleavage motif is conserved across the majority of H2A variants (Fig. S1*E*). Nonetheless, it remains to be determined if these variants are also being cleaved during differentiation or in other biological processes.

### Cathepsin L facilitates H2A proteolysis upon cellular differentiation

**Figure 2:**
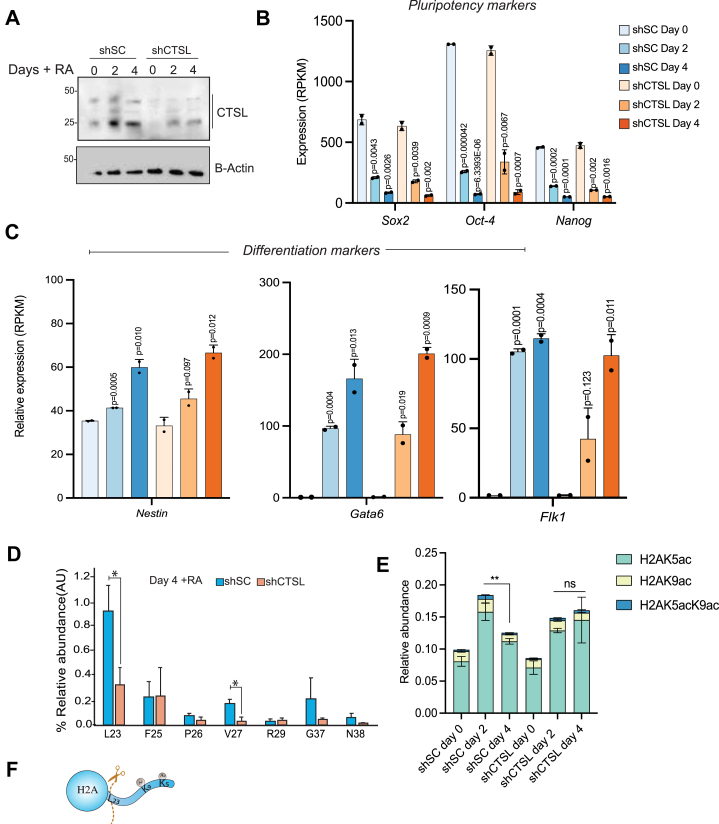
**Cathepsin L facilitates H2A proteolysis upon differentiation.***A*, validation of CTSL knockdown in undifferentiated and differentiated cells, β-Actin was used as a loading control. qPCR analysis of (*B*) pluripotency and (*C*) differentiation-associated genes in mESCs (Day 0) and embryoid bodies (Day 2 and 4) with and without shCTSL. Gene expression by RNA-seq, *bar* plots represent the average of two biological replicates, and the *error* bars represent the SD, significance was calculated using two-tailed Student's *t* test. *D*, Mass spectrometry quantification of cH2A after 4 days of RA treatment. *Asterisks* indicate significant differences between scramble shRNA (shSC) or CTSL KD cells (shCTSL), two-tail Student's *t* test (∗*p*< 0.05) and at least three biological replicates in each time point. *E*, Mass Spectrometry analysis of H2A acetylation levels during differentiation. Bars represent the average (n = 3) of the different form of acetylation across the conditions tested and different colors indicate the contribution of each acetylation mark. *Error* bars represent the S.D. and significance was calculated using two-tailed Student *t* test (∗∗*p*< 0.01). *F,* Illustration of the post translational modifications on the N-terminal tail of histone H2A.

Previously, we reported that CTSL cleaved H2A *in vitro* (21), and other studies have similarly found CTSL-mediated cleavage of histones *in vitro* using reconstituted nucleosomes (22). We therefore hypothesized that CTSL can serve as the H2A protease in cells. To test this, we knocked down the expression of CTSL using shRNA (shCTSL, shCTSL579 ad shCTSL580) in mESCs (shSC was used as a control) (Figs. 2*A* and S2*A*). Gene expression analysis by RNA-seq and qPCR showed a reduction of pluripotency markers (*Nanog*, *Oct4*, *and Sox2*) along with an increase of differentiation markers (*Nestin*, *Gata6*, *FLk1*), indicating that shCTSL mESCs are still able to differentiate (Figs. 2, *B* and *C*, S2, *B* and *C* and Data S2). To quantify the levels of cH2A upon CTSL knockdown, we again employed Top Down MS to monitor intact H2A as well as the cleavage products. We observed a significant reduction of cH2A when the expression of CTSL was reduced (Figs. 2*D* and S2, *D* and *E*). Proteolysis in the control cells was observed not only at L23 but also at other residues, including V27, suggesting that CTSL cleaves H2A at multiple sites. Our results indicate that a 50% reduction of CTSL via shRNA knockdown was sufficient to significantly reduce the levels of L23 by three-fold (Fig. 2*D*). Additionally, knockdown of Cathepsin L by shCTSL579 and shCTSL580 also decreased levels cH2A (cH2A at L23 and V27) after 4 days of RA treatment (Fig. S2*F*). *in vivo*.

To understand how cleavage of H2A by CTSL affects post-translational modification (PTM) patterns, we used high-resolution bottom-up histone PTM profiling *via* mass spectrometry (23). Our mass spectrometry data showed that the overall levels of H2A acetylation (H2AK5ac, H2AK9ac and H2K5acK9ac) oscillate during normal ESC differentiation. In undifferentiated cells, the levels of all possible forms of acetylated H2A (K5ac, K9ac and dually acetylated K5acK9ac) are together approximately 10% (Fig. 2*E*). Two days after RA treatment, acetylation levels increased significantly to 18% in shSC mESCs and up to 15% in shCTSL compared to undifferentiated ESCs. Interestingly, after 4 days of RA treatment, acetylation levels significantly decreased to 12% in shSC but not in shCTSL mESCs; in fact, the overall levels of acetylated H2A slightly but not significantly increased to 16% with shCTSL and remained unchanged with shCTSL 579 and 589 (Fig. S2*E*). Together, our data indicate that CTSL-mediated proteolysis serves to remove acetyl marks on the H2A N-terminus, thereby potentially regulating gene expression during differentiation (Fig. 2*F*).

### Knockdown of CTSL leads to genome-wide redistribution of acetylated H2A in stem cells

**Figure 3:**
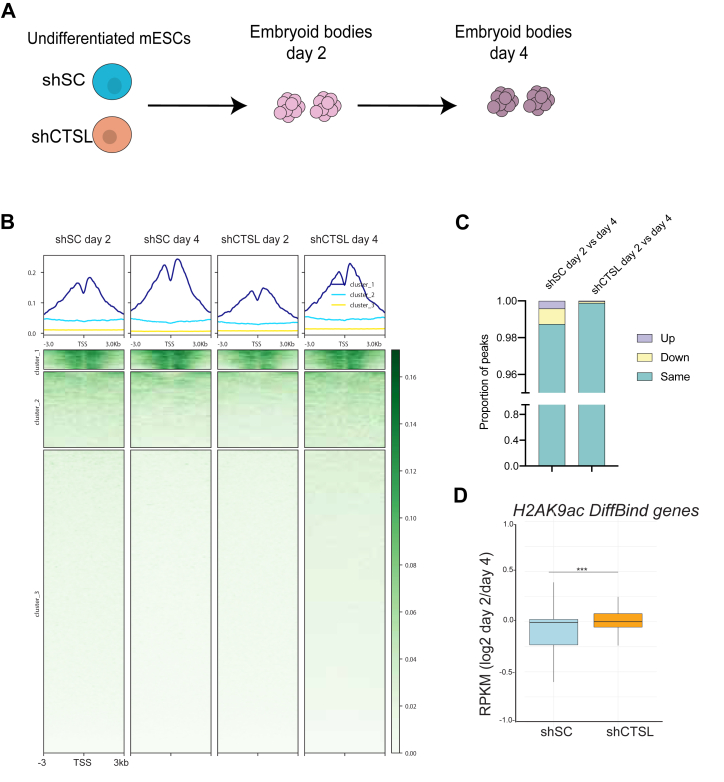
**Genome-wide localization of acetylated H2A.***A*, schematic of differentiation time points used for ChIP-seq and RNA-seq analysis. *B*, heatmap showing enrichment of H2AK9ac over mm10 genes centered around the TSS at Day 2 and Day 4 of EBs formation in control cells (shSC) or CTSL KD cells (shCTSL). *C*, DiffBind (55) analysis comparing H2AK9ac occupancy at Day 2 *versus* Day 4 in shSC and shCTSL. *D*, gene expression by RNA-seq. The boxplot indicates the log2 fold change in gene expression from Day 2 *versus* Day 4 in shSC (*blue*) and shCTSL (*orange*) using genes (n = 347) with significantly lower H2AK9ac occupancy (by DiffBind). Wilcoxon-rank sum two-sided was used to determined significance (*p* = 0.0001).

Given that histone tails, specifically those of H3 and H4, have been shown to be dynamically modified during stem cell differentiation (24), and our observations indicating that H2A is also dynamically acetylated during differentiation, we asked how proteolysis regulates the genome-wide localization of H2A acetylation. We performed ChIP-seq experiments for H2AK9ac in shSC and shCTSL mESCs, as well as in differentiated embryoid bodies (Fig. 3*A*). We focused on this mark as ChIP-seq experiments for H2AK5ac in our hands were unsuccessful. In undifferentiated cells, the total levels of H2AK9ac are ∼ 1% lower in CTSL KD cells compared to controls by MS. At the genomic level, this difference is only noted in intronic regions (Fig. S3, *A* and *B*). However, more interestingly and in agreement with our MS data, we found a genome-wide decrease of H2AK9ac on Day 2 of treatment when CTSL was knocked down, but not at Day 4 (Fig. 3*B* cluster three and Data S4). We also noticed an overall decrease in acetylation levels in several histone PTMs, including all forms of H4 acetylation (mono-, di-, tri-, and tetra-acetylation), in undifferentiated cells and at day 2 of embryoid bodies (EBs) formation, H4 mono, di, tri and tetra acetylated in undifferentiated cells and at day 2 of EBs formation (Fig. S2, *C*–*E* and Data S3) suggesting that Cathepsin L KD alters the overall histone PTMs landscape in mESCs.

Moreover, when determining the genomic distribution of H2AK9ac, we found a 4% increase at promoters in CTSL KD EBs (Fig. S3*D*). Next, we performed differential binding analysis, and here we found that ∼1.1% of peaks changed significantly between Day 2 and Day 4 in the control (Log_2_ fold change >1 (up) or < −1 (down), FDR < 0.05), which was not observed upon knockdown of CTSL (Fig. 3*C*; and Data S4). This led to the question of whether the expression of those genes is altered upon CTSL knockdown. To answer this question, we examined RNA-seq data in shSC and shCTSL cells. We hypothesized that genes that lose H2AK9ac after 4 days of RA treatment will also have lower gene expression compared to Day 2. As demonstrated in Figure 3*D* (Data S5), genes (n = 347) with significantly less H2AK9ac have lower gene expression after 4 days of differentiation in the control, but not upon CTSL knockdown. Interestingly, gene ontology (GO) analysis showed that genes with significantly decreased H2AK9ac in WT cells (n = 347) at Day 4 of RA treatment are involved in cellular differentiation and nervous system development, slightly upregulated at promoters in shCTSL cells (Fig. S3*C*). Taken together, these data suggest that H2A proteolysis by CTSL aids in gene regulation by silencing genes involved in pluripotency while activating genes to promote cell lineage commitment.

### Acetylated H2A is recognized by the PBAF complex in mESCs

**Figure 4:**
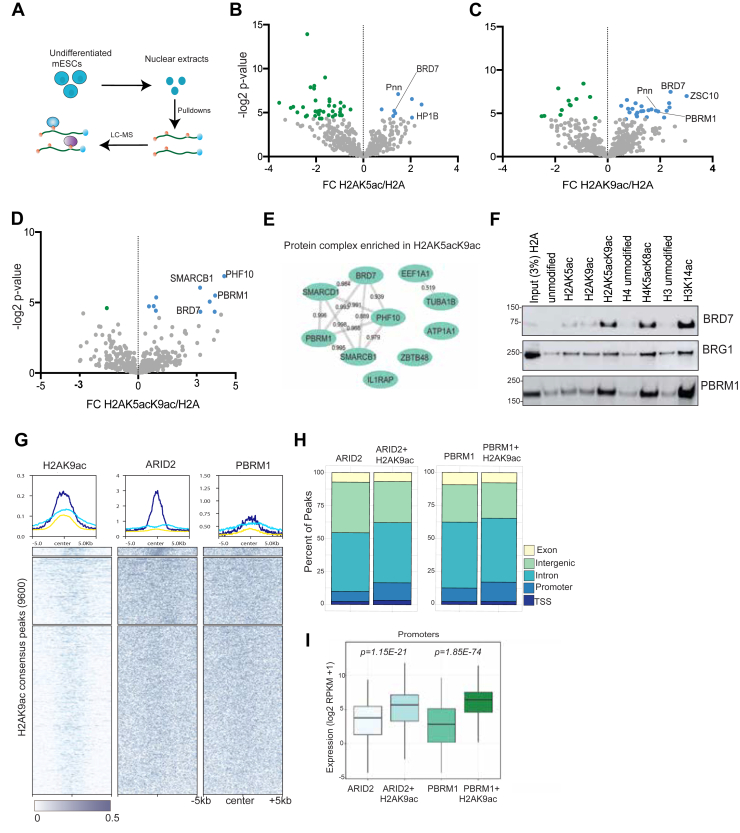
**Members of the PBAF complex bind acetylated H2A.***A*, representation of synthetic histone peptide pulldown experiments using nuclear extracts. Volcano plots showing the fold change between unmodified H2A (control) peptide and (*B*) H2AK5ac, (*C*) H2AK9ac or (*D*) H2AK5acK9ac peptides. Proteins significantly enriched by acetylated peptides *versus* the control peptide are labeled in *blue* (Student's *t* test). *E*, STRING analysis showing associations between the proteins enriched in H2AK5acK9ac pulldowns, the numbers represent the interaction strength. *F*, Western blot validation of MS results. *G*, Heatmap showing genomic enrichment of ARID2 and PBRM1 with H2AK9ac peaks in pluripotent mESCs. *H*, genome-wide distribution of PBAF components only and PBAF-H2AK9ac colocalized regions. *I*, expression levels (log_2_ RPKM+1) of promoter in PBAF components only and PBAF-H2AK9ac dual regions. Significance was determined using two-sided unpaired Student's *t* test.

Changes in the H2A acetylation patterns upon loss of CTSL expression could alter the recruitment of regulatory proteins to chromatin. In addition to H2AK9ac, our MS data showed increased levels of H2AK5ac in differentiated cells upon CTSL KD (Fig. 2*E*)). This mark is associated with gene activation (23, 25), but its reader protein remains to be identified. Thus, to identify potential readers, we employed synthetic peptides corresponding to H2AK5ac, H2AK9ac and H2AK5acK9ac as bait in peptide pulldown assays. Isolated proteins were characterized by Bottom-Up LC-MS/MS with further validation by immunoblot (Fig. 4*A*). As a negative control, we used an H2A peptide containing only N-terminal acetylation, as H2A is known to be co-translationally acetylated at the N-terminal serine residue by NatD (26, 27). All modified H2A peptides were also N-terminally acetylated as well.

The H2AK5ac and H2AK9ac peptide baits succeeded in capturing members of the Polybromo-1 Associating Factor (PBAF) chromatin remodeler complex, including BRD7 and PBRM1 (Fig. 4, *B*–*E*; and Data S6). Similarly, the dually acetylated H2AK5acK9ac was also able to pull down PBAF members, such as BRD7, SMARCB1, PHF10, PBRM11 and BRD7. Enrichment of PBAF proteins was more prominent when H2A was acetylated at K5 and K9 simultaneously (Figs. 4*D*, S4*A*). In similar experiments but with Western blots, the bromodomain-containing protein BRD7 seems to bind H2A acetylated peptides, especially the dually acetylated H2AK5acK9ac peptide, although it can also bind H3 and H4 acetylated peptides as well (Fig. 4*F*). Furthermore, we also determined that BRG1 showed similar affinity for all acetylated peptides (Figs. 4*F* and S4*A*). Since histone H4 is also known to be acetylated at its N-terminal tail, which resembles the sequence of H2A, we reasoned that acetylated H4 may also interact with the PBAF complex. Repeating our affinity pulldown experiments using unmodified and dually acetylated (K5acK8ac) H4 peptides, we observed enrichment of PBAF compared to an unmodified control (Figs. 4*F* and S4*B*). However, as expected, the main reader for H4K5acK8ac, BRD4 (24, 28), attained greater enrichment than BRD7 (Fig. S4*B*), but equal to BRG1 (Fig. S4*C*). PBRM1 has been previously reported to bind H3K14ac (29), which we likewise observed in our experiments. Furthermore, we found that PBRM1 also binds to H2AK5acK9ac (Fig. 4*F*). Additionally, we observed that TBP binds acetylated H2A and H4, highlighting its positive correlation with gene activation (Fig. S4*D*).

Upon this potential finding that the PBAF complex may bind H2AK5acK9ac, we next performed ChIP-seq for selected PBAF-specific proteins (PBRM1 and ARID2) to establish genes that are co-occupied by H2AK9ac and PBAF in mESCs (Fig. 4*G*). Our data showed that 54% of all ARID2 peaks contain H2AK9ac (n = 9257) as depicted in Figure S4*E*. Similarly, 55% of all PBRM1 peaks are also marked with H2AK9ac (n = 8316) as shown in Figure S4*F*. When we compared the PBAF occupied regions without H2AK9ac to where they coexisted, we found a remarkable increase of PBAF at gene promoters where acetylated H2A was present (Fig. 4*H*). For instance, 7.6% of peaks exclusive to ARID2 are found at the promoter; however, when acetylated H2A is present, we observe 13.3% of peaks corresponding to promoter regions. Likewise, we noticed a 4% increase in PBRM1 localization at promoters in the presence of acetylated H2A, suggesting that H2AK9ac may facilitate PBAF localization to promoters. To further correlate H2AK9ac and PBAF co-occupancy, we compared the expression of genes containing H2AK9ac and the PBAF complex at their promoters to those exclusively found for PBAF. As shown in Figure 4*I*, expression of genes that are co-occupied by PBAF and acetylated H2A have significantly higher expression than those only marked by PBAF (*p*-value < 0.0001), suggesting that potential recruitment of PBAF is enhanced by H2AK9ac and promotes gene expression.

### H2A proteolysis prevents PBAF recognition of acetylated H2A

**Figure 5:**
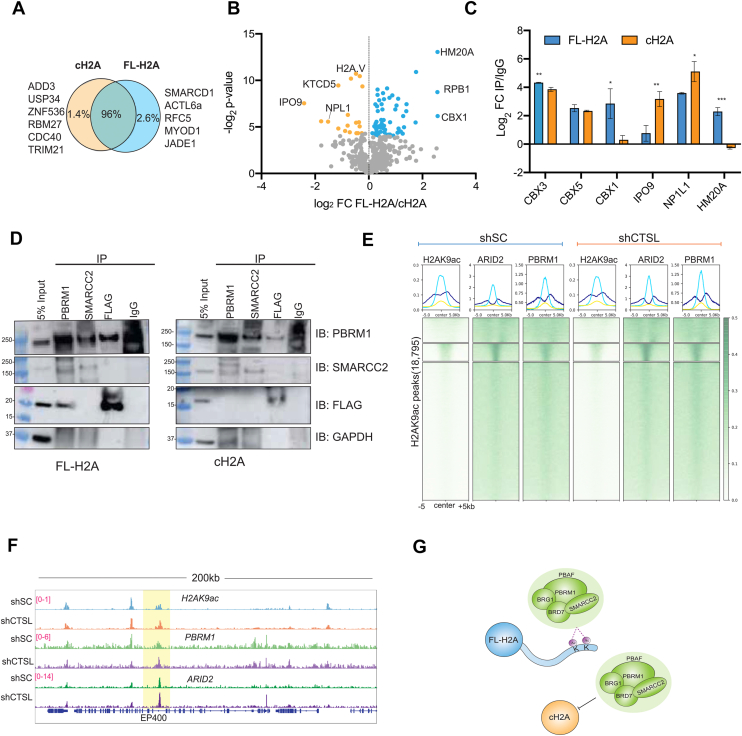
**H2A proteolysis prevents PBAF recognition of acetylated H2A.***A*, *Venn diagrams* showing overlapping and unique proteins identified by IP-MS from FL-H2A and cH2A pulldowns. *B*, *Volcano plots* showing the fold change between FL-H2A (*blue*) and cH2A (*orange*) Significantly enriched proteins by Student's *t* test are highlighted in *blue* or *orange*. Only proteins significantly enriched over IgG (FC > 2 and *p*< 0.05) were used for analysis. *C*, Bar plot of proteins differentially enriched in each condition (FL-H2A (*blue*) and cH2A (*orange*)), showing the average of three biological replicates, Student's *t* test; ∗*p*< 0.05, ∗∗*p*< 0.01, ∗∗∗*p*< 0.001. The error bars represent the SD. *D*, co-IPs using whole cell extracts of mESCs expressing either FLAG-tagged full-length H2A (FL-H2A) or FLAG-tagged cH2A (cH2A). *E*, heatmap showing enrichment of ARID2 and PBRM1 on H2AK9ac peaks in shSC and shCTSL after 4 days of RA treatment. *F*, genome browser snapshot of H2AK9ac, ARID2, and PRBM1 over EP400. *G*, Simple model depicting how acetylation on full length H2A (FL-H2A) can anchor the PBAF complex to chromatin, while this interaction is abolished in clipped H2A (cH2A).

Since modifications on histone tails can regulate the recruitment of proteins to chromatin, proteolytic severing of the tails could preclude these interactions normally mediated by modifications on the tails. We hypothesized that removal of the H2A tail, and therefore H2A tail acetylation, would disrupt binding of PBAF to H2A. To test this, we performed co-immunoprecipitation followed by MS (IP-MS) analysis using mESCs expressing either FLAG-tagged full-length H2A (FL-H2A) or N-terminally truncated H2A, representing cleavage at L23 (cH2A). Inspection of the interactome of FL-H2A compared to cH2A showed 96% of the proteins interacted with both cH2A and FL H2A (Fig. 5*A*; and Supplementary Data 7). However, in agreement with our previous findings, members of the PBAF chromatin remodeler complex (SMARCD1 and ACTL6a) were only found to interact with FL-H2A and not cH2A. Additionally, we found that Importin-9 (Ipo9) was enriched in cH2A sample compared to FL-H2A (Fig. 5, *B* and *C*). Ipo9 is known to translocate H2A-H2B dimers from the cytosol to the cell nucleus (30). We also found that NPL1 (nucleosome assembly protein 1, also known as Nap1), which is known to exchange H2A/H2B dimers (31), was enriched by cH2A over FL-H2A as well. Interestingly, we found CBX1, 3 and 5 to preferentially interact with FL-H2A (Fig. 5, *B* and *C*; and Data S6). Gene ontology analysis showed that the proteins enriched by cH2A are typically associated with RNA splicing and gene expression, while those enriched with FL-H2A are involved in nucleosome assembly and DNA packaging (Fig. S5, *A* and *B*).

We next validated our IP-MS results by doing co-immunoprecipitation assays followed by immunoblotting. As shown in Figures 5*D* and S5*C*, FL-H2A co-precipitated with PBRM1 more so than cH2A. Additionally, PBRM1 but not SMARCC2 (Baf170) co-precipitated with FL-H2A, suggesting that PBRM1 might interact with H2A acetylation through its bromodomains, which bind to acetylated lysine residues. Given that proteolysis removes the binding substrate for PBAF, we hypothesized that Cathepsin L KD cells should have higher PBAF occupancy at H2AK9ac sites. To test this hypothesis, we focused on Day 4 as cH2A abundance is at its maximum. We performed ChIP-seq for PBRM1 and ARID2 in shSC and shCTSL cells at Day 4 of RA treatment. When we analyzed the occupancy of PBAF at H2K9ac peaks, we noticed an increased occupancy of both ARID2 and PBRM1 in shCTSL KD cells (Fig. 5, *E* and *F*; and Data S8), indicating that PBAF remains bound to acetylated H2A when CTSL levels are reduced. Taken together, these results indicate that the PBAF protein complex recognizes acetylated H2A and that H2A proteolysis can abrogate this recognition.

### cH2A is associated with marks of active transcription and faster turnover

**Figure 6:**
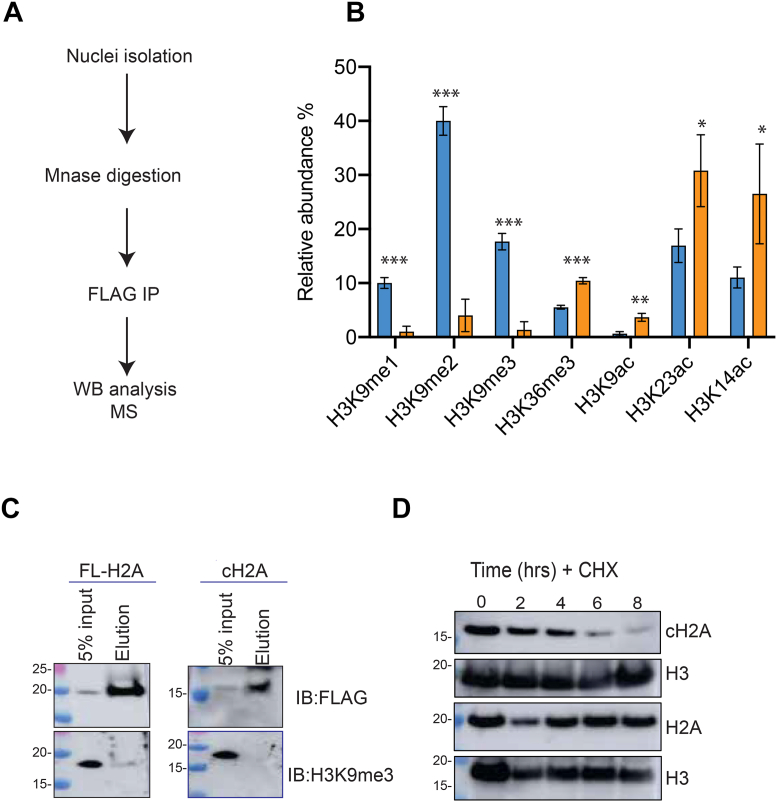
**cH2A is associated with marks of active transcription and faster turnover.***A*, Mononucleosome IP-qMS quantification of histone PTMs enriched with FL-H2A (*blue bars*) or cH2A (*orange bars*). Significance is denoted by asterisk (∗) using Student's *t* test; ∗*p*< 0.05, ∗∗*p*< 0.01, ∗∗∗*p*< 0.001. *B*, Western blot validation of H3K9me3 from MS data. *C*, cH2A and FL-H2A protein stability assay using cycloheximide (CHX 100 ng/ml) D.

Our IP-MS results found a reduced interaction between CBX proteins and cH2A. CBX1, also known as heterochromatin protein 1, plays an important role in gene silencing through its interaction with methylated H3K9 (32). This suggests that nucleosomes enriched with cH2A should also be depleted of histone H3K9me marks. To examine this possibility, we used FLAG-tagged cH2A and FL-H2A to purify mononucleosomes containing analyzed their PTMs in cis by qMS. As expected, cH2A nucleosomes were mostly depleted of mono-, di- and tri-methylation at H3K9 compared to those containing FL-H2A (Fig. 6, *A* and *B* and Data S9). The qMS were much more conclusive than the Western blots, presumably due to the enhanced specificity and sensitivity of our qMS histone profiling platform. In contrast, we found that cH2A-containing nucleosomes were associated with higher levels of histone marks associated with gene expression, such as histone H3 acetylation (at K9, K14, and K23) and H3K36me3 (Fig. 6*A*). Taken together, this data suggests that FL-H2A can co-exist with H3K9me in certain parts of the genome, while cH2A is most likely to be temporarily associated with open accessible chromatin, where its precursor full-length H2A, is hyperacetylated, possibly on promoters of active genes.

Aside from affecting histone modifications, cleavage of H2A by CTSL could also influence nucleosome structure and stability. Using hydrogen deuterium exchange coupled with mass spectrometry (HDX-MS), we have shown that in a nucleosome context, the N-terminal tail of H2A is protected from some amide exchange (33), which is consistent with the finding that histone tail proteolysis can destabilize nucleosomes *in vitro* (34). The loss of the N-terminal tail of H2A likely disrupts DNA-histone interactions that play important roles in the maintenance of nucleosome structure. Indeed, during cellular processes such as replication and transcription, the nucleosome undergoes conformational changes to allow access to the underlying DNA. Interestingly, we found that cH2A preferentially interacts with Npl1 (Fig. 5, *B* and *C*), a protein known to exchange H2A/H2B dimers. Thus, we hypothesized that cH2A-containing nucleosomes are more rapidly exchanged or evicted compared to FL-H2A-containing nucleosomes. To measure the stability of cH2A in cells, we blocked protein synthesis with cycloheximide (Figs. 6 and S6). We found that while FL-H2A protein levels remain stable for at least 8 h, cH2A undergoes a more rapid degradation within 4 to 8 h (Fig. 6*C*). While biochemical assays such as thermal shift assays or hydrogen deuterium exchange coupled with mass spectrometry using *in vitro* reconstituted nucleosomes may provide more data to support our hypothesis, our *in vivo* data showed that cH2A-containing nucleosomes are less stable than FL-H2A in mESCs. Notably, in our IP-MS experiment, we found that cH2A, but not FL-H2A interacted with Trim21 (Fig. 5*A*), a ubiquitin ligase known to target proteins for proteasomal degradation (35). To investigate whether cH2A was being degraded through the proteasome pathway, cells were treated with the proteasome inhibitor bortezomib and cH2A stability was subsequently monitored for 8 h. As shown in Figure S6*D*, the levels of cH2A still decrease after bortezomib treatment. This was further confirmed by treating cells simultaneously with cycloheximide and bortezomib (Fig. S6*E*), indicating that cH2A may be degraded by another cellular degradation pathway independently of Trim21 or could exchange in a dimer-bound form by Npl11 (a cH2A interactor) but not degraded right away. Overall, our data suggest that histone proteolysis occurs in more open chromatin regions to remove key histone PTM sites and disrupt binding interactions, thus facilitating nucleosome destabilization and eviction, while promoting gene silencing of pluripotency-related genes.

## Discussion

Histone proteins undergo a wide variety of changes to their PTM patterns to regulate gene expression. The addition of these chemical moieties, predominantly on lysine and arginine residues, serves to modulate electrostatic interaction between histones and DNA or recruit transcriptional regulators, such as chromatin remodeling complexes that can evict or slide nucleosomes along the DNA. Compared to classical histone PTMs, such as acetylation and methylation, the connection between histone proteolysis and gene expression has been well-established. The first identification of histone clipping was reported 3 decades ago, involving cleavage of histone H1 *Tetrahymena* micronuclei (36). Since then, histone proteolysis has been noted in additional organisms such as sea urchin (37), chicken (38) and yeast (39). These studies contribute to the idea that histone proteolysis represents a mechanism of gene regulation similar to canonical histone PTMs. Indeed, Duarte *et al.* found that histone H3 proteolysis during cellular senescence promotes silencing of cell cycle-related genes (16).

In the context of cellular development, CTSL has been shown to cleave histone H3 (14), but the downstream effects of this cleavage event remain unclear. Building on that prior study, we now report that histone H2A is also cleaved by CTSL during embryonic stem cell differentiation. Using high-resolution mass spectrometry, we demonstrate that H2A undergoes proteolysis at amino acid L23, accounting for almost 1% of the entire H2A population in differentiated cells (Fig. 1*D*). Although we found other cleavage sites in undifferentiated cells, they were not responsive to CTSL knockdown, which suggests the possibility of other H2A proteases beyond CTSL. In addition to cleavage at L23, we also found a less abundant secondary cleavage site at V27, which is significantly reduced upon CTSL knockdown (Fig. 2*D*). This was not surprising given that on the histone H3 tail, CTSL is known to target a primary site (A21), however, secondary cleavage sites exist. One possible explanation is that CTSL first cleaves at L23, located right before the alpha 1-helix, and then proceeds to cleave again at V27 (within the alpha one helix, further destabilizing the nucleosome by weakening DNA-histone interactions).

*In vitro* studies have found that CTSL is more active when H3 is acetylated at H3K18ac. However, it was found that dual acetylation at K18 and K23 has the opposite effect on CTSL activity (14). Given that histone H2A is also acetylated within its N-terminal tail, we sought to interrogate how proteolysis affects H2A acetylation *in vivo.* We anticipated that changes in acetylation due to proteolysis would be difficult to monitor due to the stoichiometry of the H2A clipped form being quite low (only ∼1%). Using high resolution qMS (40) we found that the overall level of acetylated H2A do not decrease after 4 days of differentiation compared to control cells (Fig. 2*E*).

It is important to reiterate that the overall abundance of H2AK5ac is roughly 8% in undifferentiated cells, which doubles 2 days after RA exposure (15%). By Day 4, the level of this mark decreases to ∼9%. A similar trend is observed with H2AK9ac, which increases from 1% in undifferentiated cells to 2% in early differentiation before decreasing back to 1% in late differentiation. These observations were preserved upon CTSL knockdown with shCTSL579/580 (Fig. S2*G*). we observed highly concordant relative distributions of histone post-translational modifications. While absolute PTM abundances differed, the proportional relationships among marks were preserved. This pattern is consistent with global scaling of histone modification levels, which can arise from differences in total histone content, chromatin accessibility, or peptide recovery inherent to MS-based analysis, rather than changes in cell-state composition.

Interestingly, we found that suppressing the levels of CTSL led to a global decrease in histone acetylation (Fig. S3, *C*–*E*) but since these observations are at the global level, we reasoned that localizing these changes more precisely in the genome may aid in uncovering how H2A acetylation regulates gene expression during cellular development. Furthermore, investigating how CTSL proteolysis affects the genome-wide distribution of acetylated H2A would elucidate how this novel process contributes to cell fate decisions through modulation of gene expression patterns. We hypothesized that genes regulated by H2A proteolysis would retain more H2A acetylation after 4 days of differentiation and concurrent knockdown of Cathepsin L. We performed ChIP-seq in control and CTSL KD mESCs. Since several attempts to perform ChIP-seq for H2AK5ac resulted in a high background and failed, we solely had to focus on the H2AK9ac mark. Surprisingly, we found a redistribution in the genomic localization of H2AK9ac. After 4 days of RA treatment, the proportion of H2AK9ac peaks in promoter regions slightly increased (Fig. 3*B* cluster 3). Examining the expression of genes with significantly lower occupancy of H2AK9ac after 4 days of differentiation compared to Day 2, we observed higher levels of gene expression upon CTSL KD. This suggests that proteolysis promotes gene silencing as expected. In line with the findings from Duarte *et al.* (16), we propose that proteolysis could serve as a rapid mechanism to silence genes involved in pluripotency, allowing cells to proceed through differentiation.

Our data indicate that H2A acetylation plays a key role in driving gene expression during development. Prior studies have found that H2A acetylation is associated with gene activation in *Drosophila* (41). Acetylation on H2AK5ac and H2AK9ac is known to be deposited by Tip60 (Kat5) (42). In ESCs, Tip60 is also known to activate genes involved in proliferation and cell renewal (43). While the role of this acetyltransferase has been extensively described, little is known about H2A acetylation in ESCs and the potential reader of this mark. Our data showed that members of the PBAF SWI/SNF chromatin remodeler complex recognized different forms of acetylated H2A and had a higher affinity for the dual acetyl mark, H2AK5acK9ac (Fig. 4). As shown in Figure 4, PBAF members also seem to bind to H4K5acK8ac as well. This might be explained by the similar sequence homology between the N-terminal tails of H4 and H2A. Consistent with prior findings, we confirmed that BRD4, the main reader of H4K5acK8ac, does not prefer to bind H2AK5acK9ac (Fig. S4*B*). As previously reported, we also observed H3K14ac to interact with BRD7 and PBRM1 (30). A possible explanation is that H3K14ac and acetylated H2A may co-exist in the same nucleosomes or in hyperacetylated chromatin regions. Yet, we cannot conclude that the interaction of the PBAF complex is mediated directly by PBRM. We also cannot exclude the possibility that PBRM1 could accommodate and bind all acetyl sites, given its six tandem bromodomains. Despite this possibility, our ChIP-Seq experiments further support our initial observation of PBAF binding to or being associated with acetylated H2A. As demonstrated in Figure 4*G*, we uncover a genome-wide interaction between PBAF and H2AK9ac. Furthermore, we noticed an increased occupancy of PBAF at promoters that are occupied by H2AK9ac (Fig. 4*H*), and these co-occupied genes have higher expression levels compared to those only occupied by PBAF (Fig. 4*I*). Together, our results suggest that H2AK9ac promotes gene expression by helping to recruit PBAF to these promoter regions.

**Figure 7:**
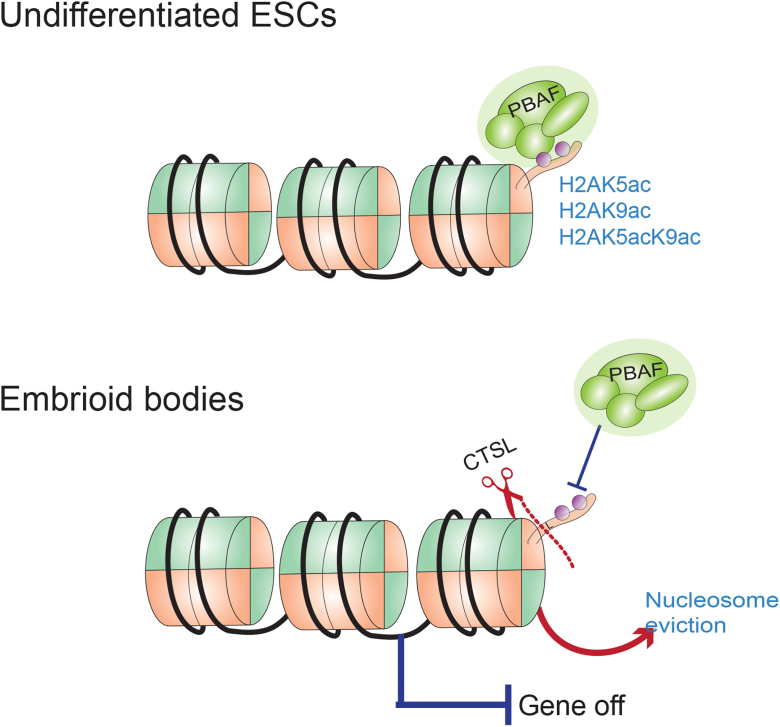
Proposed model of gene regulation by H2A proteolysis.

The PBAF complex has been shown to play a key role in maintaining pluripotency (44). In addition to H3K14ac (28), members of the BAF family are also known to interact with H3K4me1 (45). Notably, CTSL has also been shown to cleave H3 during ES differentiation (14), however, the regulatory function of H3 clipping in mESCs has not been established. Additionally, the extent to which H3K14ac and H4K5acK8ac (which also bind PBAF complex member BRD7, Fig. 4*F*) overlap with H2K9ac genome-wide is yet to be uncovered. The true reader of H4K5acK8ac is most likely BRD4, based on our data (Fig. S4*B*) and prior studies. Additionally, our results show that BRD7 prefers acetylated H2A (H2AK5ac) over the similar sequence provided by H4K5acK8ac (Fig. S4*B*). We proposed that this interaction would be diminished upon H2A proteolysis. Indeed, utilizing immuno-affinity precipitation followed by MS (IP-MS) and validation by immunoblotting, we found that FL-H2A, but not cH2A is able to interact with the PBAF complex (Fig. 5*A*). We further supported this hypothesis by performing ChIP-seq in shCTSL EBs (Day 4). Here we observed increased occupancy of PBAF members at H2AK9ac sites when we knockdown CTSL. Additionally, we also found that CBX proteins interact preferentially with FL-H2A over cH2A. Interactions between CBX proteins and FL-H2A could be mediated through the association of FL-H2A with H3K9 methylation. As demonstrated monucleosome IP'ed coupled to qMS (Fig. 6), FL-H2A-containing nucleosomes show enrichment for H3K9 methylation. Conversely, cH2A-enriched nucleosomes were found to be more associated with gene active marks, such as H3K9ac and H3K14ac, suggesting that histone proteolysis occurs in euchromatic regions. Finally, we also found that cH2A is less stable than FL H2A (Fig. 6*C*), suggesting that proteolysis may destabilize the cH2A-containing nucleosomes *in vivo*. ,÷. Taken together, our studies uncover a novel mechanism of gene regulation during cell fate determination involving cleavage of histone H2A. We propose a model (Fig. 7) in which CTSL mediates the proteolysis of H2A to remove acetylation on the N-terminal tail, thereby silencing highly specific genes involved in the maintenance of pluripotency, which are spatially and temporarily regulated. This silencing is presumably accomplished by preventing the recruitment of PBAF to acetylated H2A-containing nucleosomes, which abrogates chromatin remodeling at these genes and blocks the binding of transcriptional machinery.

## Experimental procedures

### Cell Culture

CCE-Nanog-GFP mESCs were donated by Ihor Lemischka (Mt. Sinai). Cells were grown at 37 °C in 5% CO_2_ using DMEM with high glucose and sodium pyruvate (Thermo), supplemented with 15% characterized FBS (Hyclone), non-essential amino acids (MEM) (Thermo), GlutaMax (Thermo), 10 μM 2-mercaptoethanol (Thermo) and LIF(Millipore). Cells were grown 0.1% gelatin (Millipore) coated plates, and GFP+ cells were sorted by flow cytometry before downstream experiments. For differentiation into embryoid bodies formation, ∼1.5 × 10^7^cells were plated in 10-cm dishes in media without LIF and supplemented with 10 μM all-trans retinoic acid (Sigma). Cells were kept with constant rotation for 4 days and the media was changed every other day. All cell lines were tested for *mycoplasma*.

### Plasmids, cloning, and generation of stable cell lines

To generate CTSL KD and Flag-tagged H2A (FL and cH2A) mESCs, approximately 4 × 10^5^ HEK293T cells were transfected with packaging vectors pPAX2 and VSGV (kind gifts from Dr Shelley Berger, UPenn) along with 1 μg of FL-H2A or cH2A, or shRNA targeting CTSL. shRNA plasmid for CTSL KD (in pLKO.1) was obtained from the high-throughput core at Penn, and scramble control was a gift from Dr Shelly Berger (UPenn). To generate FLAG-Tagged FL-H2A and cH2A tagged with FLAG at the C-terminus, DNA fragments were cloned into pLenti plasmid (Origene) (EcoRI and PspXI). HEK293T were transfected in mESCs media (without LIF) and virus particles were collected for 3 days. Prior to transduction, the viral supernatant was filtered using a 0.45 μm filter. Polybrene (Santa Cruz Biotechnology) was added to a final concentration of 8 ug/ml. Cells were infected for 24 h followed by selection with 1 μg/ml of puromycin (Thermo) for 5 days. Transduction efficiency of FL-H2A and cH2A was validated by immunoblotting with FLAG antibody (Sigma). CTSL knockdown efficiency was confirmed by RT-qPCR and immunoblots, with GAPDH and β-actin used as controls. To assess protein stability, mESCs expressing FL-H2A and cH2A were treated with 100 μg/ml cycloheximide (Sigma), 10 nM Bortezomib (Sigma) or a combination of both drugs for 8 h with time points collected every 2 h. Cell pellets were snap frozen in liquid nitrogen and stored at −80 °C before use.

### Histone isolation and nuclear extraction

Histones were isolated as previously described (19). In summary, after nuclei isolation, histones were acid extracted with H_2_SO_4_ for 3 h, followed by precipitation with trichloroacetic acid (TCA) overnight. The resulting pellets were washed first with acetone containing 0.1% of hydrochloric acid (HCl), followed by a wash in acetone alone. Finally, samples were resuspended in ultrapure water and protein concentrations were determined by Bradford assay (BioRad). To generate nuclear extracts, ∼1 × 10^6^cells were lysed in hypotonic buffer (10 mM Tris pH 8, 2 mM MgCl_2_, 24 mM CaCl_2_, 0.3 M sucrose, 0.5% NP-40 and protease inhibitors) for 5 min on ice. The nuclei were pelleted at 500g for 5 min and washed with PBS. Nuclear proteins were released on ice for 30 min in nuclear extraction buffer (10 mM HEPES pH 7.9, 0.75 mM MgCl_2_, 500 mM NaCl, 0.1 mM EDTA, 12.5% glycerol) supplemented with 2.5 U/ul benzonase (Millipore) and protease inhibitors (Bimake).

### Antibodies

All antibodies used in this study are listed in Table S1.

### Co-immunoprecipitation (Co-IPs) and protein identification by mass spectrometry

Co-IPs were performed as previously described (46). Briefly, 2 × 10^7^cells were lysed for 1 h at 4 °C in IP buffer (20 mM Tris pH 7.5, 137 mM NaCl, 1 mM CaCl_2_, 1% NP-40, 10% glycerol, 1 mM MgCl_2_ and 12U/ml Benzonase) supplemented with protease inhibitors (Bimake). Antibodies (FLAG, BAF180, BAF170, IgG) were conjugated to 30 μl protein G magnetic beads (Thermo) in blocking solution (0.5% BSA in PBS) for at least 2 h at 4 °C. Cell lysate (∼600 μg) was added to the conjugated beads and incubated overnight at 4 °C. IP samples were washed three times with IP buffer and eluted in SDS loading buffer for immunoblots or processed for MS analysis by on-beads digestion (FL-H2A and cH2A IPs) as follows. Before trypsin digestion, beads were resuspended in 50 mM NH_4_HCO_3_, and reduction with DTT, alkylation with iodoacetamide and digested with Trypsin (Promega). Resulting peptides were dried by SpeedVac centrifugation and stored at −80 °C. For MS analysis, samples were resuspended in MS buffer A (0.1% formic acid in MS-grade water) and analyzed by LC-MS. Samples were loaded onto a 17-cm in-house C_18_ column (Reprosil-Pur) using a Dionex LC (Thermo Scientific) coupled to a QE-HF orbitrap mass spectrometer (Thermo Scientific). Peptides were eluted over a 90-min gradient from 5 to 35% solvent B (solvent A: 0.1%FA, solvent B: 80% acetonitrile, 0.1% FA), the flow rate was set to 300 nl/min. Data were obtained in data-dependent acquisition (DDA) mode. The MS1 was acquired over 300 to 1200*m/z* with a resolution of 60,000, AGC target of 5e5, and maximum injection time of 100 ms. For the MS2, the top 25 most intense ions were selected for MS/MS by high-energy collision dissociation (HCD) at 27 NCE, with a resolution of 30,000, AGC target of 1e5, and maximum injection time of 150 ms. Raw files were processed using MaxQuant (v 1.6.0.16) (47) using *M. Musculus* database (Uniprot, April 2017). For MS/MS database searches, the precursor mass tolerance was set to 4.5 ppm and the product mass tolerance to 0.5 Da. Two trypsin missed cleavages were allowed. Carbamidomethyl (C) was set as a static modification while oxidation (M) and acetylation (protein N-terminus) were selected as variable modifications. Proteins were quantified using label-free quantification (iBAQ). The protein false discovery rate (FDR) was filtered for < 0.01.

### Mononucleosme IP and histone PTMs analysis by mass spectrometry

Cells were lysed in Buffer A (10 mM HEPES pH 7.9, 10 mM KCL, 1.5 mM MgCl_2_, 0.34 M sucrose, 10% glycerol, 1 mM DTT, 0.1% Triton-X and protease inhibitors) on ice for 5 min. After centrifugation, pellets were washed with Buffer A without Triton-X. Soluble nuclear proteins were released for 15 min on ice in no salt buffer (3 mM EDTA, 0.2 mM EGTA, 0.1 mM DTT). Mononucleosomes were obtained by digesting the chromatin in 30 units of Micrococcal nuclease (Mnase) (Roche) in digestion buffer (50 mM HEPES, 2 mM CaCl_2,_ 0.2% NP-40) for 10 min at 37 °C. Chromatin was briefly sonicated at 20% amplitude. Samples were further clarified by centrifugation, and DNA was examined by agarose gel electrophoresis. Monucleosomes were dialyzed against Buffer D (20 mM HEPES pH 7.9, 20% glycerol, 0.2 mM EDTA, 0.2% Triton X, and protease inhibitors) for 2 h at 4 °C. Following dialysis, ∼100ug of mononucleosomes were incubated with FLAG-M2 beads (20 μl) overnight at 4 °C. After three washes with Buffer D (Buffer A plus 100 mM NaCl), samples were eluted with 3X FLAG peptide (0.33 mg/ml) on ice for 45 min and then analyzed by immunoblotting and MS. For MS analysis, samples were run on an SDS-PAGE gel, and bands between 10 and 25 kDa were excised. Histone peptides were derivatized with propionic anhydride as previously described (48). In summary, after two rounds of in gel derivatization, samples were digested with 12.5 ng/μl of trypsin (Promega) overnight. Peptides were eluted form the gel after several iterations of hydration with 50 mM NH_4_HCO_3_ and dehydration with 100% acetonitrile. Samples were dried by SpeedVac centrifugation and resuspended in 50 mM NH_4_HCO_3_ for two more rounds of derivatization. Peptides were desalted and analyzed by LC-MS described above. Peptides were eluted over a 60-min gradient from 5 to 35% solvent B (A:0.1% FA, B:80% acetonitrile, 0.1% FA), with a flow rate of 300 nl/min. Histone MS data were obtained by data-independent acquisition (DIA). The MS1 was acquired in high resolution (120,000) from 300-1100*m/z*. For MS2, 16 DIA scans with 50*m/z* windows were acquired using HCD (NCE = 35). Raw files were processed using our in-house developed software EpiProfile (49). These experiments were performed in triplicate.

### Peptide pulldowns and protein identification by MS

Histone peptide pulldown assays were performed as previously described (50) with minor modifications. In brief, nuclear extracts were prepared by lysing mESC in buffer A (10 mM HEPES pH 7.9, 1.5 mM MgCl_2_, 10 mM KCl) supplemented with protease inhibitors. Cells were incubated on ice for 10 min and pelleted at 400*g* for 5 min. Subsequently, cell pellets were further homogenized in buffer A supplemented with 0.15% NP-40 and protease inhibitors. After nuclei were pelleted after spinning at 3200*g* for 15 min, nuclear proteins were released for 1 h at 4 °C in Buffer C (420 mM NaCl, 20 mM HEPES pH 7.9, 20% glycerol, 2 mM MgCl_2_, 0.1% NP-40 and protease inhibitors). Protein concentration was determined by Bradford assay.

Synthetic peptides were purchased from GenScript. Peptides were designed to include H2AK5ac, H2AK9ac, H2AK5acK9ac, H3K14ac and H4K5acK8ac, as well as corresponding control “unmodified” peptides without internal lysine acetylation. All peptides were biotinylated at the C-terminus. The exact sequences of the peptides are listed in Table S2. Each peptide (∼25ug) was bound to 75 μl of Dynabeads MyOne C1 (ThermoFisher) for 20 min at room temperature in peptide binding buffer (150 mM NaCl, 50 mM Tris pH 8, 0.15 %NP-40). Nuclear lysate was diluted to 0.6 μg/ul in protein binding buffer (150 mM NaCl, 50 mM Tris pH 8, 0.15% NP-40, 0.5 mM DTT, 10 uM ZnCl_2_ and protease inhibitors). Nuclear lysates (∼500ug) were incubated with conjugated peptides overnight at 4 °C. After 5 washes with wash buffer (protein binding + 350 mM NaCl) samples were eluted with SDS loading dye for MS or immunoblot analysis. For MS analysis, samples were resolved by SDS-PAGE (in 4–12% NuPage). Lanes were cut from 10 to 250 kDa and divided into 4 different fractions. Before trypsin digestion, proteins were reduced with 5 mM DTT for 45 min at 55 °C and alkylated with 55 mM iodoacetamide at room temperature for 30 min. Samples were digested overnight, and peptides were extracted as previously mentioned above. Peptides were identified by MS as described in the Co-IPs section.

### Top-Down analysis of intact H2A by LC-MS

Prior to mass spectrometry analysis, individual histones were fractionated by reversed-phase liquid chromatography (RP-HPLC) as previously described (51). Briefly, ∼200 μg of purified histones were fractionated using Vydac C18 column (4.6 mm inner diameter, 250 mm length and 5 μM particle size). Samples were eluted using a 100 min linear gradient (30–60%B). Solvent A consisted of 5% acetonitrile (ACN) in HPLC-grade water and 0.2% trifluoracetic acid (TFA), where Buffer B was 95% ACN and 0.2% TFA. Fractions corresponding to H2A.1 were collected based on known retention time and characteristic peaks (Fig. S1*A*). Samples were then dried using a SpeedVac and stored at −80 °C. Fractions corresponding to H2A.1 were resuspended in 0.1% formic acid (FA) to a final concentration of 1 μg/μl. Samples (2 μg) were loaded onto a 75-um (inner diameter) × 20 -cm C18 column (Reprosil-Pur, Germany) for nano-LC. Proteoforms were eluted over 60 min using a non-linear gradient (10–40B%). Solvent A consisted of 0.1% formic acid, while solvent B was 80% acetonitrile (ACN). nLC was coupled to a hybrid Orbitrap Fusion Mass Spectrometer equipped with electron transfer dissociation (ETD) fragmentation (Themo). The acquisition was done in data-dependent mode with a 3 s cycle time and using high resolution of MS1 (120,000 at 200 m/z) and tandem MS2 (30,000). For MS1 and MS2, the AGC target was set to 2.0E5 with a max injection time of 100 ms for MS1 and 300 ms for MS2. Charge states 5 to 21 were selected for MS2 fragmentation with ETD; finally, three micro scans were averaged to yield high-resolution MS/MS spectra. Data analysis was performed as described previously with minor modifications (52). In brief, after spectral deconvolution with Xtract (Thermo), files were searched with Mascot (v2.5 Matrix Science). Acetylation (K) and methylation (KR) were included as dynamic modifications. No enzyme was selected for digestion. Results files were further processed using IsoScale (52) with filtering for unambiguous identification. All cleavage sites were normalized to the abundance of the full-length H2A, manually extracted using Xcalibur (Thermo Fisher Scientific) and the Skyline program (53).

### Chromatin immunoprecipitation followed by sequencing (ChIP-seq)

ChIP-seq was performed as previously reported (46). Briefly, 1 × 10^7^ mESCs were fixed in 1% formaldehyde in PBS for 10 min and then quenched with 125 mM glycine for 5 min. Chromatin was sonicated with a S220 Focused-ultrasonicator (Covaris) for 15 min and immunoprecipitated with anti-H2AK9ac (Abcam). Antibodies were conjugated to protein G Dynabeads (Thermo). After washing and elution, samples were incubated overnight at 65 °C to reverse cross-links. DNA was purified after treatment with RNAse (Thermo) and Proteinase K (Thermo). Purified DNA was quantified using Qubit dsDNA kit (Thermo), and 50 ng was used to prepare sequencing libraries. Input samples were also included and prepared using the same protocol. NEBNext Ultra DNA library kit for Illumina (New England Biolabs) was used to prepare the libraries, and the quality was assessed by Agilent BioAnalyzer 2100 (Agilent). Quantification was performed using KAPA library quantification kits (KAPA Biosystems) or NEBNext library Quant kit (New England Biolabs). For each condition, two independent replicates were included. Single-end sequencing (75 bp) was performed on a NextSeq 500 platform. (Illumina). ARID2 and PBRM1 ChIP-seq was done as reported by Michel et. Al (54). Briefly, 2 × 10^7^ mESCs were fixed with 1% formaldehyde for 5 min and quenched for an additional 5 min. After nuclear extraction, chromatin was sonicated with a S220 Focused-ultrasonicator (Covaris) for 12 min and immunoprecipitated with anti-PBRM1 (Bethyl) and anti-ARID2 (Bethyl). After washing and elution, samples were incubated overnight at 65 °C to reverse cross-links. DNA was purified after treatment with RNAse (Thermo) and Proteinase K (Thermo). Using the Qiagen minElute PCR purification kit (Qiagen). Purified DNA was quantified using Qubit dsDNA kit (Thermo). Libraries were prepared using the KAPA Hyper Prep kit Sequencing was performed at the Genome Sequencing Facility in St Jude Children's Research Hospital on Illumina HiSeq platform in single-end mode with 50 bp per read.Reads were checked for sequencing quality with FastQC (v0.11.2). After that, reads were aligned to the mouse reference genome (mm9) using Bowtie2 (v2.2.9) with soft-clipping allowed. SAMtools (v0.1.19) was used to filter out non-primary alignments, alignments with mapping quality scores less than 10, PCR duplicates, and alignments on non-chromosome contigs or ENCODE blacklist regions. Peaks were detected for each sample against the corresponding input control using SICER (v1.1) with default parameters. Bedtools (v2.27.1) was used to generate bedgraph files for visualization on genome browser, in which each sample was normalized to 10 million reads per library with the corresponding input subtracted.

### Whole transcriptome analysis by RNA-seq

RNA was extracted utilizing an RNAeasy Kit (Qiagen) following the manufacturer's instructions. Treatment with DNase for 15 min was included to degrade all genomic DNA. Libraries were prepared using NEBNext Poly(A) mRNA magnetic isolation module and NEB Ultra Directional RNA Library kit for Illumina (New England Biolabs). Library quality, quantification and sequencing were done as described above. RNA-seq reads were checked for sequencing quality with FastQC (v0.11.2). After sequencing, reads were aligned to the mouse reference genome (mm9) using STAR (v.2.3.0e) with default parameters. SAMtools (v0.1.19) was used to filter out alignments with a mapping quality score less than 10 and alignments on mitochondria or non-chromosome contigs. Finally, FeatureCounts (v.1.6.2) was used to generate a matrix of mapped fragments per RefSeq-annotated gene. Differential gene expression analysis was performed using the DESeq2 R package (v.1.16.1) with an FDR cutoff of 0.05, and log2 fold change cutoff of 1.5X. Bedtools (v2.27.1) was used to generate bedgraph files for visualization on genome browser, in which each sample was normalized to 10 million reads per library.

### GO analysis

All gene ontologies analysis were performed using GENEONTOLOGY at http://geneontology.org/.

## Data availability

Mass spectrometry raw files are deposited in Chorus repository project number 1698. Additionally, all ChIP and RNA seq files are in the NCBI Gene Expression Omnibus under the following accession number GSE162896.

## Supporting information

This article contains supporting information.

## Conflict of interest

The authors declare that they do not have any conflicts of interest with the content of this article.

## Supporting information

Data S9

Data Summary

## References

[1] Strahl B.D., Allis C.D. (2000). The language of covalent histone modifications. Nature.

[2] Chen C.C.L., Goyal P., Karimi M.M., Abildgaard M.H., Kimura H., Lorincz M.C. (2018). H3S10ph broadly marks early-replicating domains in interphase ESCs and shows reciprocal antagonism with H3K9me2. Genome Res..

[3] Keller G. (2005). Embryonic stem cell differentiation: emergence of a new era in biology and medicine. Genes Dev..

[4] Mas G., Blanco E., Ballaré C., Sansó M., Spill Y.G., Hu D. (2018). Promoter bivalency favors an open chromatin architecture in embryonic stem cells. Nat. Genet..

[5] Dixon J.R., Jung I., Selvaraj S., Shen Y., Antosiewicz-Bourget J.E., Lee A.Y. (2015). Chromatin architecture reorganization during stem cell differentiation. Nature.

[6] Giadrossi S., Dvorkina M., Fisher A.G. (2007). Chromatin organization and differentiation in embryonic stem cell models. Curr. Opin. Genet. Dev..

[7] Atlasi Y., Stunnenberg H.G. (2017). The interplay of epigenetic marks during stem cell differentiation and development. Nat. Rev. Genet..

[8] Biran A., Meshorer E. (2012). Concise review: chromatin and genome organization in reprogramming. Stem Cells Dayt.

[9] Saraiva N.Z., Oliveira C.S., Garcia J.M. (2010). Histone acetylation and its role in embryonic stem cell differentiation. World J. Stem Cells.

[10] Glibert P., Vossaert L., Van Steendam K., Lambrecht S., Van Nieuwerburgh F., Offner F. (2014). Quantitative proteomics to characterize specific histone H2A proteolysis in chronic lymphocytic leukemia and the myeloid THP-1 cell line. Int. J. Mol. Sci..

[11] Tvardovskiy A., Wrzesinski K., Sidoli S., Fey S.J., Rogowska-Wrzesinska A., Jensen O.N. (2015). Top-down and middle-down protein analysis reveals that intact and clipped human histones differ in post-translational modification patterns. Mol. Cell. Proteomics.

[12] Liu H., Wang C., Lee S., Deng Y., Wither M., Oh S. (2017). Clipping of arginine-methylated histone tails by JMJD5 and JMJD7. Proc. Natl. Acad. Sci. U. S. A..

[13] Kim K., Punj V., Kim J.-M., Lee S., Ulmer T.S., Lu W. (2016). MMP-9 facilitates selective proteolysis of the histone H3 tail at genes necessary for proficient osteoclastogenesis. Genes Dev..

[14] Duncan E.M., Muratore-Schroeder T.L., Cook R.G., Garcia B.A., Shabanowitz J., Hunt D.F. (2008). Cathepsin L proteolytically processes histone H3 during mouse embryonic stem cell differentiation. Cell.

[15] Goulet B., Baruch A., Moon N.-S., Poirier M., Sansregret L.L., Erickson A. (2004). A cathepsin L isoform that is devoid of a signal peptide localizes to the nucleus in S phase and processes the CDP/Cux transcription factor. Mol. Cell..

[16] Duarte L.F., Young A.R.J., Wang Z., Wu H.-A., Panda T., Kou Y. (2014). Histone H3.3 and its proteolytically processed form drive a cellular senescence programme. Nat. Commun..

[17] Tholen M., Hillebrand L.E., Tholen S., Sedelmeier O., Arnold S.J., Reinheckel T. (2014). Out-of-frame start codons prevent translation of truncated nucleo-cytosolic cathepsin L in vivo. Nat. Commun..

[18] Tobin D.J., Foitzik K., Reinheckel T., Mecklenburg L., Botchkarev V.A., Peters C. (2002). The lysosomal protease cathepsin L is an important regulator of keratinocyte and melanocyte differentiation during hair follicle morphogenesis and cycling. Am. J. Pathol..

[19] Lin S., Garcia B.A. (2012). Examining histone posttranslational modification patterns by high resolution mass spectrometry. Methods Enzymol..

[20] Nashun B., Yukawa M., Liu H., Akiyama T., Aoki F. (2010). Changes in the nuclear deposition of histone H2A variants during pre-implantation development in mice. Dev. Camb. Engl..

[21] Anderson L.C., Karch K.R., Ugrin S.A., Coradin M., English A.M., Sidoli S. (2016). Analyses of histone proteoforms using front-end electron transfer dissociation-enabled orbitrap instruments. Mol. Cell. Proteomics.

[22] Papanastasiou M., Mullahoo J., DeRuff K.C., Bajrami B., Karageorgos I., Johnston S.E. (2019). Chasing tails: Cathepsin-L improves structural analysis of histones by HX-MS. Mol. Cell. Proteomics.

[23] Wang Z., Zang C., Rosenfeld J.A., Schones D.E., Barski A., Cuddapah S. (2008). Combinatorial patterns of histone acetylations and methylations in the human genome. Nat. Genet..

[24] Gonzales-Cope M., Sidoli S., Bhanu N.V., Won K.-J., Garcia B.A. (2016). Histone H4 acetylation and the epigenetic reader Brd4 are critical regulators of pluripotency in embryonic stem cells. BMC Genomics.

[25] Vavouri T., Lehner B. (2012). Human genes with CpG island promoters have a distinct transcription-associated chromatin organization. Genome Biol..

[26] Tweedie-Cullen R.Y., Brunner A.M., Grossmann J., Mohanna S., Sichau D., Nanni P. (2012). Identification of combinatorial patterns of post-translational modifications on individual histones in the mouse brain. PLoS One.

[27] Magin R.S., Liszczak G.P., Marmorstein R. (2015). The molecular basis for histone H4- and H2A-specific amino-terminal acetylation by NatD. Struct. Lond. Engl. 1993.

[28] Miller T.C.R., Simon B., Rybin V., Grötsch H., Curtet S., Khochbin S. (2016). A bromodomain–DNA interaction facilitates acetylation-dependent bivalent nucleosome recognition by the BET protein BRDT. Nat. Commun..

[29] Porter E.G., Dhiman A., Chowdhury B., Carter B.C., Lin H., Stewart J.C. (2019). PBRM1 regulates stress response in epithelial cells. iScience.

[30] Padavannil A., Sarkar P., Kim S.J., Cagatay T., Jiou J., Brautigam C.A. (2019). Importin-9 wraps around the H2A-H2B core to act as nuclear importer and histone chaperone. eLife.

[31] Chen X., D'Arcy S., Radebaugh C.A., Krzizike D.D., Giebler H.A., Huang L. (2016). Histone chaperone Nap1 is a major regulator of histone H2A-H2B dynamics at the inducible GAL locus. Mol. Cell Biol..

[32] Kaustov L., Ouyang H., Amaya M., Lemak A., Nady N., Duan S. (2011). Recognition and specificity determinants of the human Cbx chromodomains. J. Biol. Chem..

[33] Karch K.R., Coradin M., Zandarashvili L., Kan Z.-Y., Gerace M., Englander S.W. (2018). Hydrogen-deuterium exchange coupled to Top- and middle-down mass spectrometry reveals histone tail dynamics before and after nucleosome assembly. Struct. Lond. Engl. 1993.

[34] Nurse N.P., Jimenez-Useche I., Smith I.T., Yuan C. (2013). Clipping of flexible tails of histones H3 and H4 affects the structure and dynamics of the nucleosome. Biophys. J..

[35] Clift D., So C., McEwan W.A., James L.C., Schuh M. (2018). Acute and rapid degradation of endogenous proteins by trim-away. Nat. Protoc..

[36] David Allis C., Bowen J.K., Abraham G.N., Glover C.V.C., Gorovsky M.A. (1980). Proteolytic processing of histone H3 in chromatin: a physiologically regulated event in tetrahymena micronuclei. Cell.

[37] Morin V., Sanchez-Rubio A., Aze A., Iribarren C., Fayet C., Desdevises Y. (2012). The protease degrading sperm histones post-fertilization in sea urchin eggs is a nuclear cathepsin L that is further required for embryo development. PLoS One.

[38] Mandal P., Azad G.K., Tomar R.S. (2012). Identification of a novel histone H3 specific protease activity in nuclei of chicken liver. Biochem. Biophys. Res. Commun..

[39] Santos-Rosa H., Kirmizis A., Nelson C., Bartke T., Saksouk N., Cote J. (2009). Histone H3 tail clipping regulates gene expression. Nat. Struct. Mol. Biol..

[40] Karch K.R., Sidoli S., Garcia B.A. (2016). Identification and quantification of histone PTMs using high-resolution mass spectrometry. Methods Enzymol..

[41] Doiguchi M., Nakagawa T., Imamura Y., Yoneda M., Higashi M., Kubota K. (2016). SMARCAD1 is an ATP-dependent stimulator of nucleosomal H2A acetylation via CBP, resulting in transcriptional regulation. Sci. Rep..

[42] Jacquet K., Fradet-Turcotte A., Avvakumov N., Lambert J.-P., Roques C., Pandita R.K. (2016). The TIP60 complex regulates bivalent chromatin recognition by 53BP1 through direct H4K20me binding and H2AK15 acetylation. Mol. Cell.

[43] Acharya D., Hainer S.J., Yoon Y., Wang F., Bach I., Rivera-Pérez J.A. (2017). KAT-independent gene regulation by Tip60 promotes ESC self-renewal but not pluripotency. Cell Rep.

[44] Hiramatsu H., Kobayashi K., Kobayashi K., Haraguchi T., Ino Y., Todo T. (2017). The role of the SWI/SNF chromatin remodeling complex in maintaining the stemness of glioma initiating cells. Sci. Rep.

[45] Local A., Huang H., Albuquerque C.P., Singh N., Lee A.Y., Wang W. (2018). Identification of H3K4me1-Associated proteins at mammalian enhancers. Nat. Genet..

[46] Lin-Shiao E., Lan Y., Coradin M., Anderson A., Donahue G., Simpson C.L. (2018). KMT2D regulates p63 target enhancers to coordinate epithelial homeostasis. Genes Dev..

[47] Cox J., Mann M. (2008). MaxQuant enables high peptide identification rates, individualized p.p.b.-range mass accuracies and proteome-wide protein quantification. Nat. Biotechnol..

[48] Sidoli S., Garcia B.A. (2017). Characterization of individual histone posttranslational modifications and their combinatorial patterns by mass spectrometry-based proteomics strategies. Methods Mol. Biol..

[49] Yuan Z.-F., Sidoli S., Marchione D.M., Simithy J., Janssen K.A., Szurgot M.R. (2018). EpiProfile 2.0: a computational platform for processing epi-proteomics mass spectrometry data. J. Proteome Res..

[50] Vermeulen M. (2012). Identifying chromatin readers using a SILAC-based histone peptide pull-down approach. Methods Enzymol..

[51] Greer S.M., Sidoli S., Coradin M., Schack Jespersen M., Schwämmle V., Jensen O.N. (2018). Extensive characterization of heavily modified histone tails by 193 nm ultraviolet photodissociation mass spectrometry via a middle–down strategy. Anal. Chem..

[52] Sidoli S., Lu C., Coradin M., Wang X., Karch K.R., Ruminowicz C. (2017). Metabolic labeling in middle-down proteomics allows for investigation of the dynamics of the histone code. Epigenetics Chromatin.

[53] MacLean B., Tomazela D.M., Shulman N., Chambers M., Finney G.L., Frewen B. (2010). Skyline: an open source document editor for creating and analyzing targeted proteomics experiments. Bioinforma. Oxf. Engl..

[54] Michel B.C., D'Avino A.R., Cassel S.H., Mashtalir N., McKenzie Z.M., McBride M.J. (2018). A non-canonical SWI/SNF complex is a synthetic lethal target in cancers driven by BAF complex perturbation. Nat. Cell Biol..

[55] Stark R., Brown G. (2023).

