# Supplementary material for: Cleavage of histone H2A during embryonic stem cell differentiation destabilizes nucleosomes to counteract gene activation
Source: J Biol Chem. 2026 Apr 9;302(6):111437. doi: 10.1016/j.jbc.2026.111437 (PMC13156748; doi:10.1016/j.jbc.2026.111437)

Supplementary figure 1

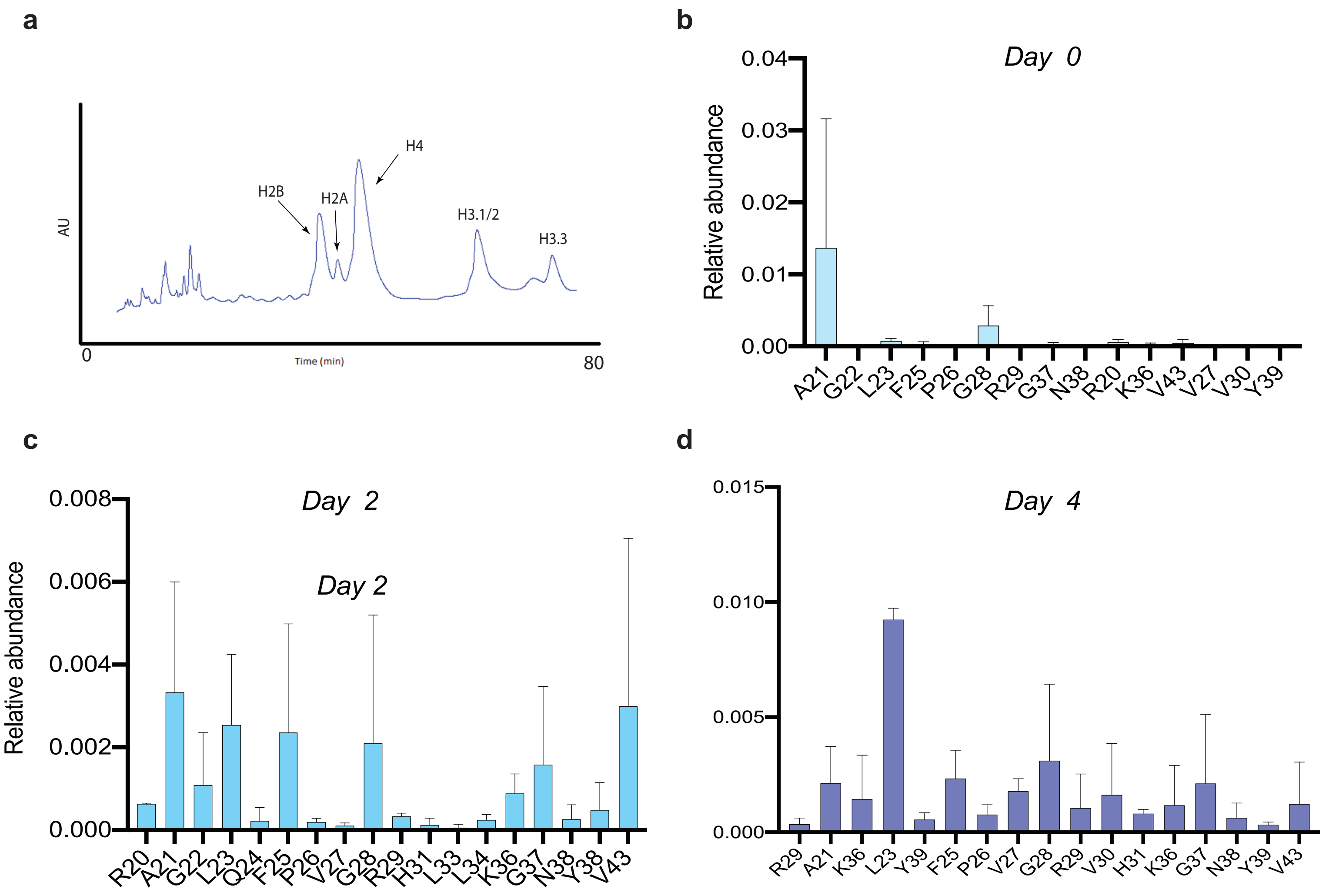

e

H2A2A_MOUSE MSGRGKQGGKARAKAKSRSSRAGLQFPVGRVHRLLRKGN-YAERVGAGAPVYMAAVLE
H2A1H_MOUSE MSGRGKQGGKARAKAKTRSSRAGLQFPVGRVHRLLRKGN-YSERVGAGAPVYLAAVLE
H2AX_MOUSE MSGRGKTGGKARAKAKSRSSRAGLQFPVGRVHRLLRKGH-YAERVGAGAPVYLAAVLE
H2A3_MOUSE MSGRGKQGGKARAKAKSRSSRAGLQFPVGRVHRLLRKGN-YSERVGAGAPVYLAAVLE
H2AB1_MOUSE --- MARKRQRRRRRKVTRSQRAELQFPVSRVDRFLREGN-YSRRLSSSAPVFLAGVLE
H2A1K_MOUSE MSGRGKQGGKARAKAKTRSSRAGLQFPVGRVHRLLRKGN-YSERVGAGAPVYLAAVLE
H2AY_MOUSE ---MSSRGGKKKSTKTSRSAKAGVIFPVGRMLRYIKKGH-PKYRIGVGAPVYMAAVLEYLTAE
H2AZ_MOUSE GGKAGKDSGKAKTKAVSRSQRAGLQFPVGRIHRHLKSRTTSHGRVGATAAVYSAAILE
H2A1C_MOUSE MSGRGKQGGKARAKAKTRSSRAGLQFPVGRVHRLLRKGN-YSERVGAGAPVYLAAVLE
H2A1O_MOUSE MSGRGKQGGKARAKAKTRSSRAGLQFPVGRVHRLLRKGN-YSERVGAGAPVYLAAVLE

Supplementary figure 2

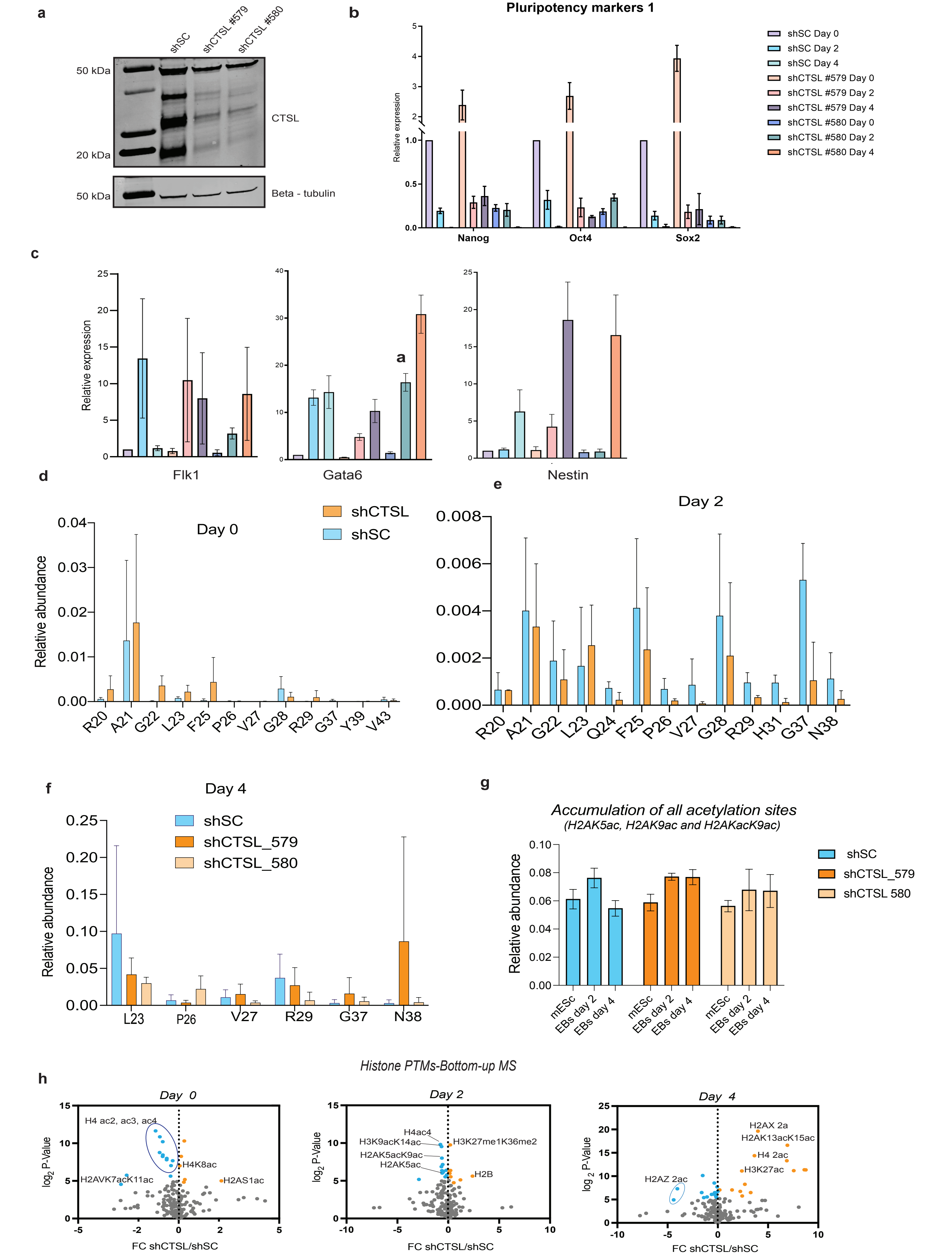

Supplementary figure 3

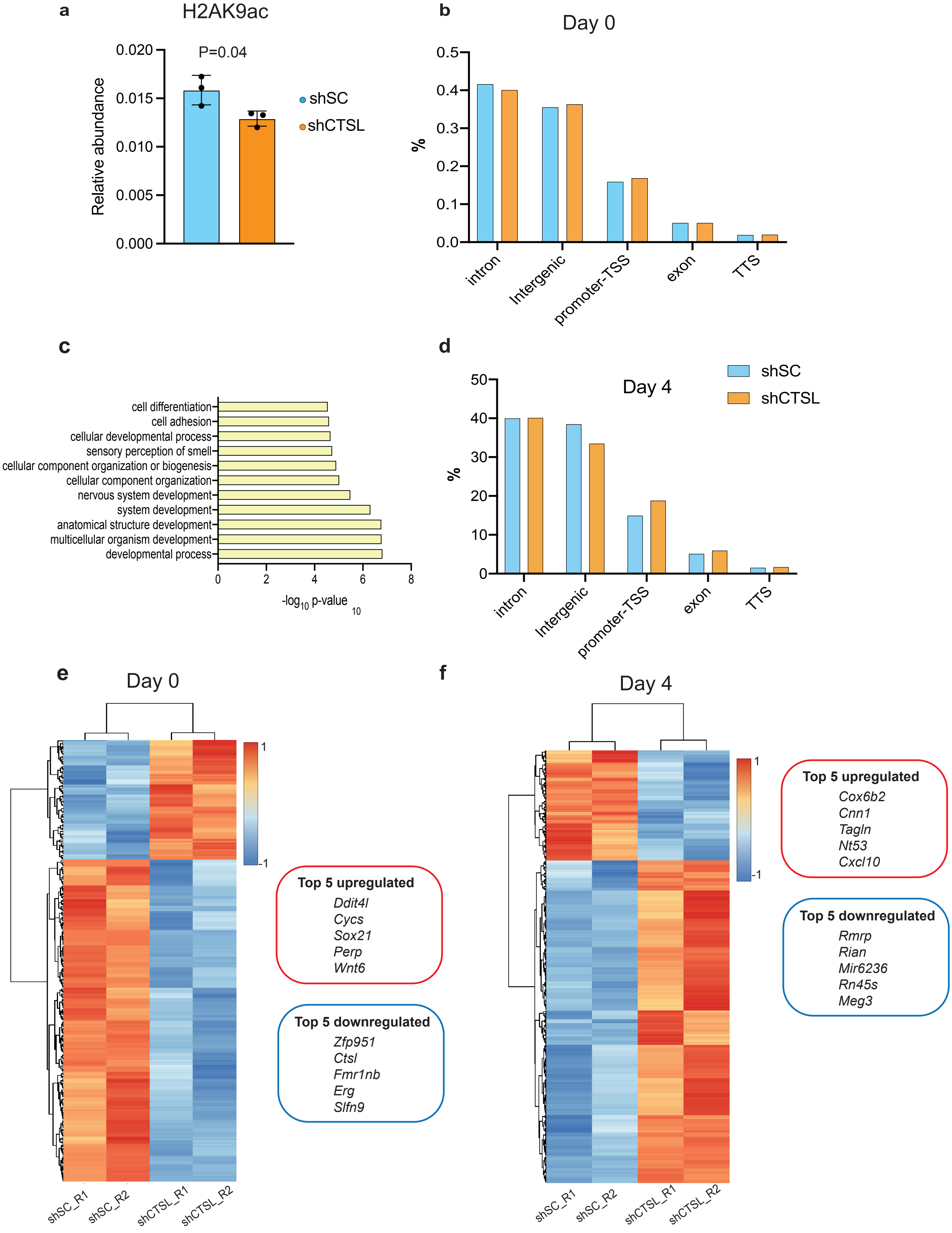

Supplementary figure 4

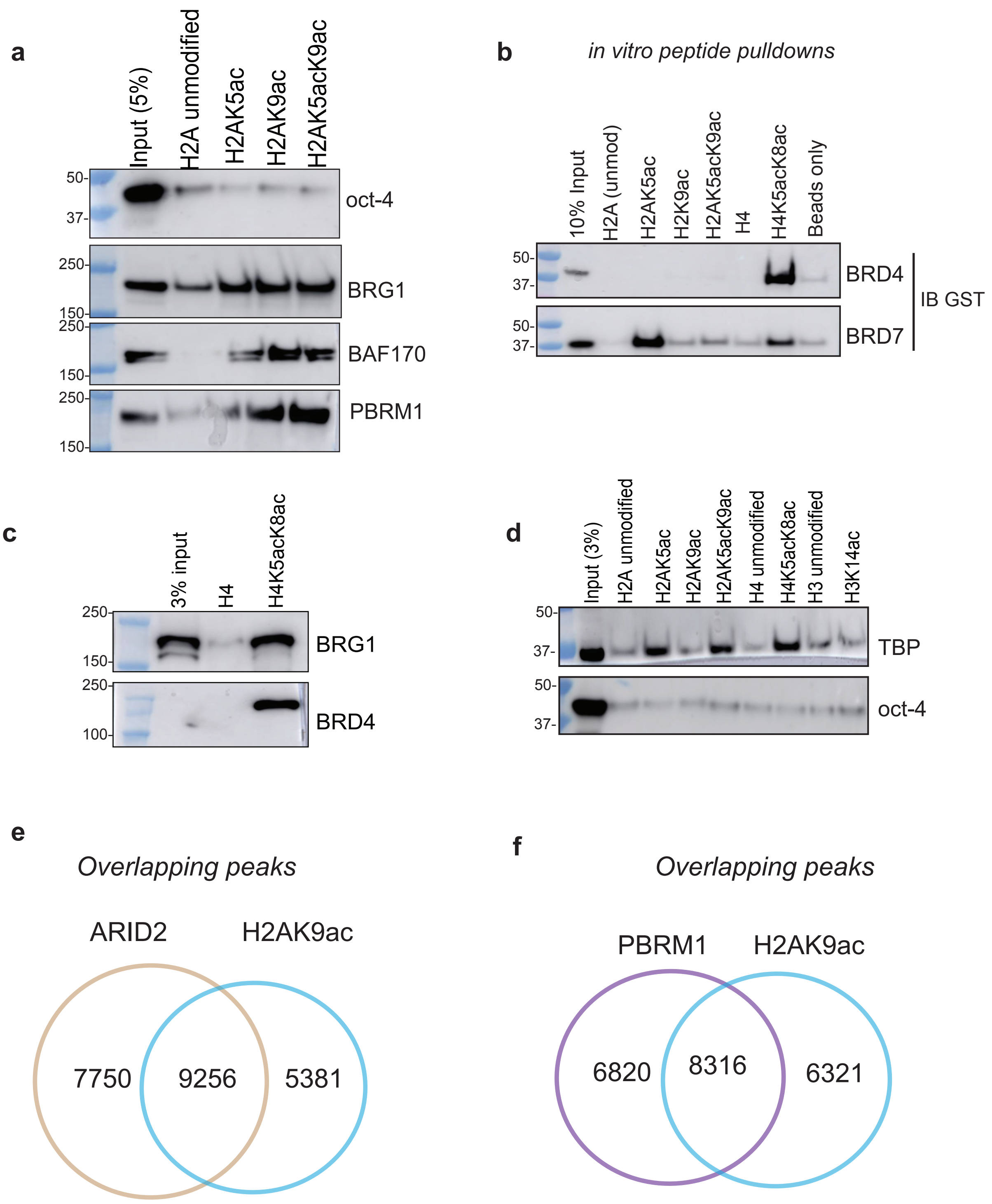

Supplementary figure 5

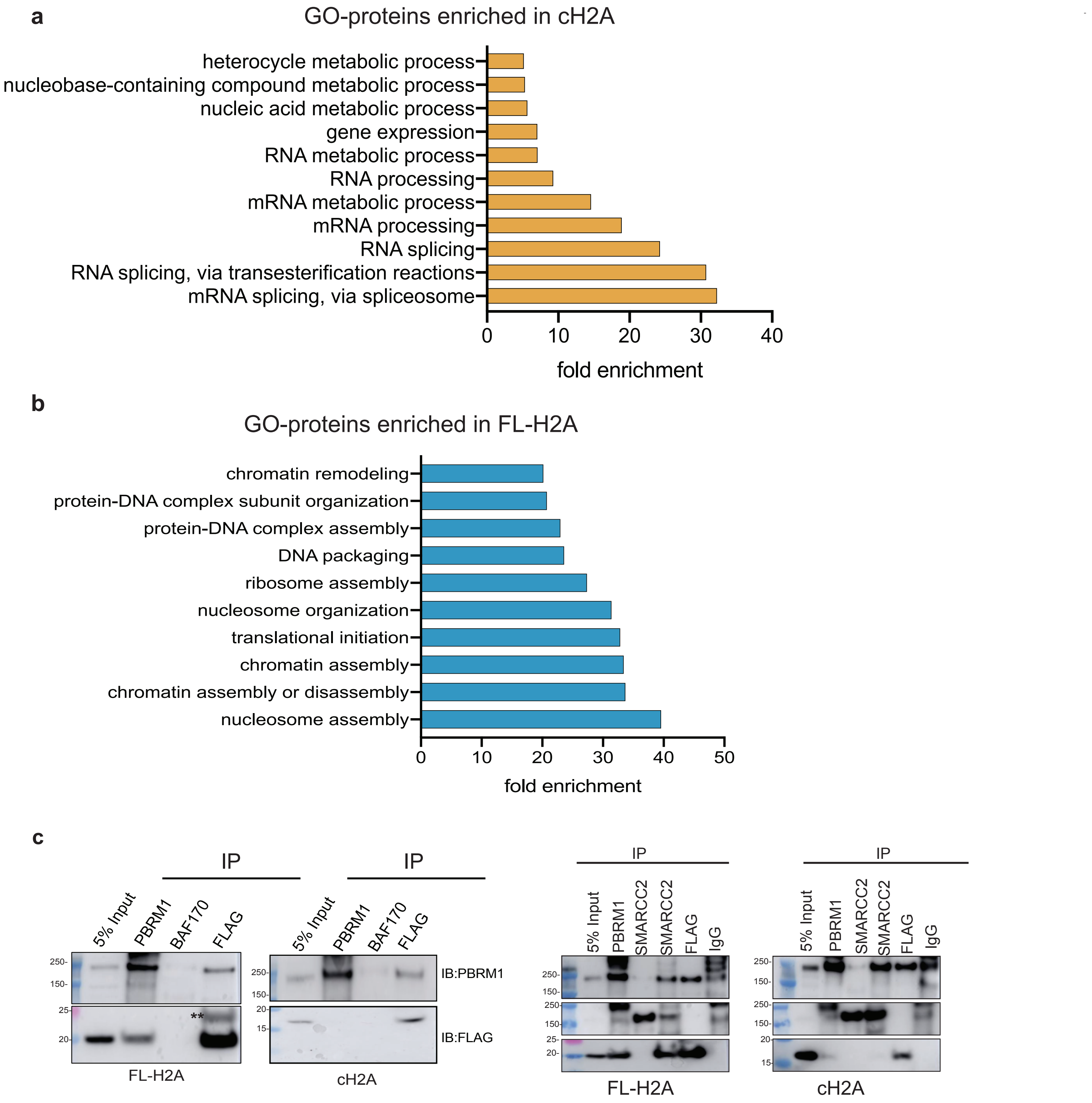

Supplementary figure 6

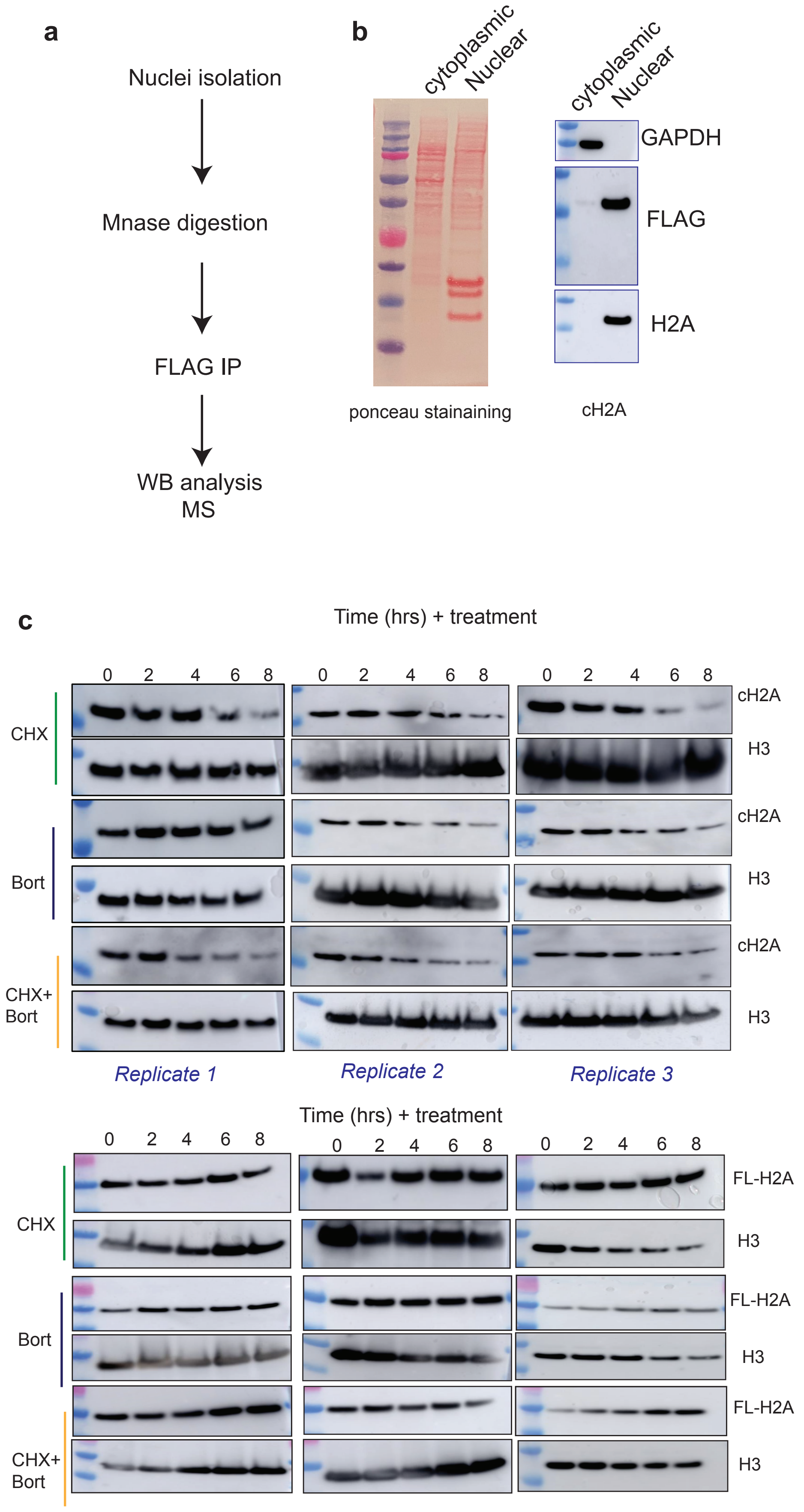

Supplement: Figures S1–S6 [file mmc1.pdf]
