# Supplementary material for: Cleavage of histone H2A during embryonic stem cell differentiation destabilizes nucleosomes to counteract gene activation
Source: J Biol Chem. 2026 Apr 9;302(6):111437. doi: 10.1016/j.jbc.2026.111437 (PMC13156748; doi:10.1016/j.jbc.2026.111437)
Supplement: Table S1 [file mmc2.docx]

**Supplementary Table 2. Synthetic peptides used in this study (20aa)**

| **Histone** | **Peptide sequence** | **Name** |
| --- | --- | --- |
| H2A | Ac-SGRGKQGGKARAKA{Lys(Biotin)}TRSSR | H2A unmodified |
|  | Ac-SGRG{Lys-Ac}QGGKARAKA{Lys(Biotin)}TRSSR | H2AK5ac |
|  | Ac-SGRGKQGG{Lys-Ac}ARAKA{Lys(Biotin)}TRSSR | H2AK9ac |
|  | Ac-SGRG{Lys-Ac}QGG{Lys-Ac}ARAKA{Lys(Biotin)}TRSSR | H2AK5acK9ac |
| H4 | Ac-SGRGKGGKGLGKGGAKRHR{Lys(Biotin)} | H4 unmodified |
|  | Ac-SGRG{Lys-Ac}GG{LysAc}GLGKGGAKRHR{Lys(Biotin)} | H4K5acK8ac |
| H3 | ARTKQTARKSTGGKAPR{Lys(Biotin)}QL | H3 unmodified |
|  | ARTKQTARKSTGG{Lys-Ac}APR{Lys(Biotin)}QL | H3K14ac |
