# Supplementary material for: Cleavage of histone H2A during embryonic stem cell differentiation destabilizes nucleosomes to counteract gene activation
Source: J Biol Chem. 2026 Apr 9;302(6):111437. doi: 10.1016/j.jbc.2026.111437 (PMC13156748; doi:10.1016/j.jbc.2026.111437)
Supplement: Table S2 [file mmc3.docx]

**Supplementary Table 1. Antibodies used in this study.**

| **Target** | **Source** | **Concentration/Application** |
| --- | --- | --- |
| FLAG | Sigma F3165 | 3ug (Ip), 1:5000 WB |
| BAF180  BAF180 | Millipore ABE70  Bethyl A700-019 | 5ug (Ip), 1:1000 WB  5ug ChIP |
| BAF170  BAF170 | Active motif 61471 (IP)  Cell signaling 12760 (WB) | 5ug (Ip)  1:1000 WB |
| ARID2  ARID2 | Thermo Invitrogen pa5-5128  Bethyl A302-230A (ChIP-seq) | 1:1000 WB  5ug ChIP |
| BRG1 | Abcam 110641 | 1:1000 WB |
| BRD7 | Cell signaling 15125s | 1:1000 WB |
| TBP | Abcam 133239 | 1:1000 WB |
| OCT4 | Abcam 19857 | 1:1000 WB |
| H2AK9ac | Abcam 177312 | 3ug ChIP |
| H2A-acidic patch | Millipore 07-146 | 1:1000 WB |
| Cathepsin L | R&D biosystems AF1515 | 1:1000 WB |
| GAPDH | Cell signaling 97166 | 1:10000 WB |
| BRD4 | Abcam 128874 | 1:1000 WB |
| GST | Cell signaling 2622s | 1:1000 WB |
| H3 | Abcam 1791 | 1:5000 WB |
| H2B | Cell Signaling 12364 | 1:1000 WB |
| IgG | Abcam 171870 | 5ug (Ip) |
| B-Actin | Cell Signaling 5125 | 1:10000 WB |
