# Supplementary material for: Cleavage of histone H2A during embryonic stem cell differentiation destabilizes nucleosomes to counteract gene activation
Source: J Biol Chem. 2026 Apr 9;302(6):111437. doi: 10.1016/j.jbc.2026.111437 (PMC13156748; doi:10.1016/j.jbc.2026.111437)
Supplement: Supplementary Figure — legends [file mmc14.docx]

**Supplementary Figure legends**

**Supplementary Figure 1. A.** Representative chromatogram of reverse-phase fractionation (RP-HPLC) of acid-extracted histones from mESCs. Top Down quantification of proteolytic events found at **B.** Day 0, **C.** Day 2 and **D.** Day 4. Error bars represent standard deviations of 3 experiments.  **E.** Sequence alignment of H2A variants, yellow box shows sequence homology on the most abundant cleavage site of H2A after 4 days of differentiation.

**Supplementary Figure 2** (**A)** Western blot confirming Cathepsin L knockdown using shRNAs 579 and 580; β-tubulin was used as a loading control. **(B)** RT–qPCR analysis of pluripotency markers (Nanog, Oct4, and Sox2) and **(C)** differentiation markers (Flk1, Gata6, and Nestin). **(D–F)** Top-down mass spectrometry quantification of cleaved H2A (cH2A) in undifferentiated mESCs and after two days (D–E) or four days **(F)** of RA-induced differentiation in cells expressing shCTSL 579 or 580 (n = 3). (**G**) Bottom-up MS analysis of N-terminal H2A acetylation marks. Bar plots represent the sum of all acetylated forms (H2AK5ac, H2AK9ac, and H2AK5acK9ac); error bars indicate the standard deviation of at least three biological replicates. (**H)** EB formation. In the volcano plots, the x-axis shows the log2 fold change of three biological replicates, while the y-axis represents the log2 P-value.

**Supplementary Figure 3.  A.** MS quantification of H2AK9ac in undifferentiated shSC control (blue) or shCTSL treated cells (orange).  using synthetic peptides as standards. Error bars represent standard deviation of 3 experiments. **B.** Distribution of H2AK9ac peaks across genomic features in undifferentiated cells shSC control (blue) and shCTSL cells (orange). ChIP-seq of undifferentiated cells performed once. **CD.** Gene ontology of differentially downregulated genes from Day 2 to Day 4 of EBs.**CD..** Genomic distribution of H2AK9ac on Day 4 of EBs formation in control shSC or after shCTSL cells. RNA-seq analysis showing significantly regulated genes (log_2_ fold change >0.5 and p-value >0.05) upon CTSL KD in undifferentiated mESCs (E) and at day four of EBs formation (F). Two biological replicates were included in this analysis.   **D.** Gene ontology of differentially downregulated genes from Day 2 to Day 4 of EBs.

**Supplementary Figure 4.  A.** Peptide pull-down experiments using unmodified H2A, H2AK5ac, H2AK9ac or H2K5acK9ac peptides. Pull-downs were carried out using nuclear extracts of undifferentiated ESCs. Western blots were performed against proteins that showed >2-fold enrichment by MS. **B.** *In vitro* peptide pull-down using recombinant BRD4 or recombinant BRD7. **C.** Peptide pull-downs of unmodified H4 and H4K5acK8ac. Western blot against BRG1 and BRD4, well-known reader of acetylated H4. **D.** Peptide pull-downs using unmodified H2A, H2K5ac, K9ac, H2K5acK9ac, H3, H3K14ac, H4 and H4K5acK8ac peptides. Western blots were performed against Oct4 (negative control) and TBP. Venn diagrams showing overlap of **E.** ARID2 peaks containing H2AK9ac, and **F.** PBRM1 peaks containing with H2AK9ac

**Supplementary Figure 5.** Gene ontology of proteins found to interact with **A.** cH2A or **B.** FL-H2A with FC > 0.5 and p-value > 0.05 using unpaired Student’s t-tests. Top 10 categories are shown. **C.** Biological replicate of Co-IPs in Figure 5D.

**Supplementary Figure 6. A.** Simplified schematic of monoucleosme IP procedure. **B.** Biochemical fractionation showing cH2A is chromatin associated. **C** Biological replicates of protein stability experiments described in Figure 6 C-E.

**Supplementary Table 1. Antibodies used in this study**

| **Target** | **Source** | **Concentration/Application** |
| --- | --- | --- |
| FLAG | Sigma F3165 | 3ug (Ip), 1:5000 WB |
| BAF180  BAF180 | Millipore ABE70  Bethyl A700-019 | 5ug (Ip), 1:1000 WB  5ug ChIP |
| BAF170  BAF170 | Active motif 61471 (IP)  Cell signaling 12760 (WB) | 5ug (Ip)  1:1000 WB |
| ARID2  ARID2 | Thermo Invitrogen pa5-5128  Bethyl A302-230A (ChIP-seq) | 1:1000 WB  5ug ChIP |
| BRG1 | Abcam 110641 | 1:1000 WB |
| BRD7 | Cell signaling 15125s | 1:1000 WB |
| TBP | Abcam 133239 | 1:1000 WB |
| OCT4 | Abcam 19857 | 1:1000 WB |
| H2AK9ac | Abcam 177312 | 3ug ChIP |
| H2A-acidic patch | Millipore 07-146 | 1:1000 WB |
| Cathepsin L | R&D biosystems AF1515 | 1:1000 WB |
| GAPDH | Cell signaling 97166 | 1:10000 WB |
| BRD4 | Abcam 128874 | 1:1000 WB |
| GST | Cell signaling 2622s | 1:1000 WB |
| H3 | Abcam 1791 | 1:5000 WB |
| H2B | Cell Signaling 12364 | 1:1000 WB |
| IgG | Abcam 171870 | 5ug (Ip) |
| B-Actin | Cell Signaling 5125 | 1:10000 WB |

**Supplementary Table 2. Synthetic peptides used in this study (20aa)**

| **Histone** | **Peptide sequence** | **Name** |
| --- | --- | --- |
| H2A | Ac-SGRGKQGGKARAKA{Lys(Biotin)}TRSSR | H2A unmodified |
|  | Ac-SGRG{Lys-Ac}QGGKARAKA{Lys(Biotin)}TRSSR | H2AK5ac |
|  | Ac-SGRGKQGG{Lys-Ac}ARAKA{Lys(Biotin)}TRSSR | H2AK9ac |
|  | Ac-SGRG{Lys-Ac}QGG{Lys-Ac}ARAKA{Lys(Biotin)}TRSSR | H2AK5acK9ac |
| H4 | Ac-SGRGKGGKGLGKGGAKRHR{Lys(Biotin)} | H4 unmodified |
|  | Ac-SGRG{Lys-Ac}GG{LysAc}GLGKGGAKRHR{Lys(Biotin)} | H4K5acK8ac |
| H3 | ARTKQTARKSTGGKAPR{Lys(Biotin)}QL | H3 unmodified |
|  | ARTKQTARKSTGG{Lys-Ac}APR{Lys(Biotin)}QL | H3K14ac |

**Supplementary Table 3. Primers used in this study 3’-5.**

| Target | Forward sequence | Reverse sequence |
| --- | --- | --- |
| *Nanog* | TGATTCAGAAGGGCTCAGCA | CTTCCAGATGCGTTCACCAG |
| *Sox2* | AGTGGTACGTTAGGCGCTTC | TGGACATTTGATTGCCATGT |
| *Oct4* | GGGGCTGTATCCTTTCCTCT | GCTGGTGCCTCAGTTTGAAT |
| *Flk1* | GCTCCTGACTACACTACCCC | CCCAAATGCTCCACCAACTC |
| *Gata6* | TTCTACACAAGCGACCACCT | TTGAGGTCACTGTTCTCGGG |
| *Nestin* | GCCACTGAAAAGTTCCAGCT | AGGGACATCTTGAGGTGTGC |
| *Gapdh* | AGGTCGGTGTGAACGGATTTG | TGTAGACCATGTAGTTGAGGTCA |
